# Pol32, an accessory subunit of DNA polymerase delta, plays an essential role in genome stability and pathogenesis of *Candida albicans*

**DOI:** 10.1080/19490976.2022.2163840

**Published:** 2023-01-05

**Authors:** Shraddheya Kumar Patel, Satya Ranjan Sahu, Bhabasha Gyanadeep Utkalaja, Swagata Bose, Narottam Acharya

**Affiliations:** a Laboratory of Genomic Instability and Diseases, Department of Infectious Disease Biology, Institute of Life Sciences, Bhubaneswar, India; b Regional center of Biotechnology, Faridabad, India; c School of Biotechnology, Kalinga Institute of Industrial Technology, Bhubaneswar, India

**Keywords:** DNA replication, genomics, PCNA, DNA polymerase delta, *candida*, candidiasis, Hsp90, azoles, drug resistance, virulence

## Abstract

*Candida albicans* is a pathobiont that inflicts serious bloodstream fungal infections in individuals with compromised immunity and gut dysbiosis. Genomic diversity in the form of copy number alteration, ploidy variation, and loss of heterozygosity as an adaptive mechanism to adverse environments is frequently observed in *C. albicans*. Such genomic variations also confer a varied degree of fungal virulence and drug resistance, yet the factors propelling these are not completely understood. DNA polymerase delta (Polδ) is an essential replicative DNA polymerase in the eukaryotic cell and is yet to be characterized in *C. albicans*. Therefore, this study was designed to gain insights into the role of Polδ, especially its non-essential subunit Pol32, in the genome plasticity and life cycle of *C. albicans*. PCNA, the DNA clamp, recruits Polδ to the replication fork for processive DNA replication. Unlike in *Saccharomyces cerevisiae*, the PCNA interaction protein (PIP) motif of CaPol32 is critical for Polδ’s activity during DNA replication. Our comparative genetic analyses and whole-genome sequencing of *POL32* proficient and deficient *C. albicans* cells revealed a critical role of Pol32 in DNA replication, cell cycle progression, and genome stability as SNPs, indels, and repeat variations were largely accumulated in *pol32* null strain. The loss of *pol32* in *C. albicans* conferred cell wall deformity; Hsp90 mediated azoles resistance, biofilm development, and a complete attenuation of virulence in an animal model of systemic candidiasis. Thus, although Pol32 is dispensable for cell survival, its function is essential for *C. albicans* pathogenesis; and we discuss its translational implications in antifungal drugs and whole-cell vaccine development.

## Introduction

In eukaryotes, DNA replication is coordinated by three essential DNA polymerases Polα, Polδ, and Polε. Extensive genetic analyses in *Saccharomyces cerevisiae* suggest that Polε is involved in only leading strand DNA synthesis, whereas Polδ synthesizes both leading and lagging strands of DNA.^1,2^ Polα-primase provides the RNA-DNA primer to initiate DNA replication from the origin. Both Polδ and Polε interact with the proliferating cell nuclear antigen (PCNA) sliding clamp to carry out processive DNA synthesis.^3,4^ Polδ holoenzyme from *S. cerevisiae* consists of three subunits: the catalytic Pol3 and the accessory structural Pol31 and Pol32 proteins. In humans, the corresponding subunits are POLD1, POLD2, and POLD3.^5^ In vertebrates and *Schizosaccharomyces pombe*, Polδ has one additional subunit p12/cdm1.^5,6^ Coherent with the central role of Polδ in genome replication, Pol3, and Pol31 subunits are indispensable for cell survival, whereas, in budding yeast, the Pol32 subunit is nonessential.^7^ The catalytic subunit Pol3 has both DNA synthesizing and proofreading activities, however, despite Pol31 being essential, its exact function in Polδ holoenzyme has not been demonstrated except that it bridges Pol3 and Pol32 subunits. Interestingly, the Pol32 homologue Cdc27 in *S. pombe* is indispensable, suggesting its organism-specific importance in DNA replication.^8^ The *pol32* null strain of *S. cerevisiae* exhibits severe defects in DNA replication, repair, and mutagenesis.^7^ Moreover, the combination of *pol32Δ* with conditional mutations in *POL3, POL31*, or *POL30* (PCNA) causes synthetic lethality. Surprisingly, deletion analyses of ScPol32 suggest that the phenotypes exhibited by *pol32Δ* cells are due to the essential role of the N-terminal domain that is involved in binding to Pol31 or Pol1 subunit of Polα, but not due to the C-terminal tail that harbors the PCNA interaction protein motif (PIP). Thus, it was assumed that the role of Pol32 is to stabilize the interaction between Pol3 and Pol31 proteins. Recent reports also suggest the Pol31-Pol32 subcomplex is a part of Polζ holoenzyme, a mutagenic translesion DNA synthesis (TLS) polymerase.^9,10^ Therefore, the damage-induced mutagenesis gets reduced significantly in *POL32* deficient cells.^11,12^ However, in DT40 cells, it was shown that the reduction in UV or MMS induced mutagenesis due to the lack of *POLD3* is mostly Polζ independent.^13^ Further, the study reported that Polδ promotes the bypass of an abasic site only when POLD3 is present. Thus, Pol32 could possess different species-specific functions. In our earlier study, we have shown that PIP motifs in all three subunits contribute to PCNA-stimulated DNA synthesis by ScPolδ, and mutational inactivation of all three PIP motifs abrogates its ability to synthesize DNA in the presence of PCNA.^3^ Genetic analyses further revealed that in the absence of functional Pol32 PIP, PCNA binding by both Pol3 and Pol31 becomes essential for cell viability. Contrarily, the cryoEM structures of the Polδ-PCNA-DNA complex both from *S. cerevisiae* and humans suggest that only Pol3/POLD1 protein binds PCNA.^14,15^ Moreover, the orientation of the Pol31 and Pol32 subcomplex in the Polδ structure is such that it prevents the interaction of the subcomplex with the DNA template as well, despite Pol31 harboring a DNA binding oligonucleotide binding (OB) fold. Thus, the precise role of Pol31 and Pol32 subunits in an eukaryotic cell remains a mystery.

Polδ has not been characterized yet in the pathogenic yeast *Candida albicans*, a causative agent of candidiasis. Despite existing antifungal arsenals, the mortality rate of systemic candidiasis caused by *C. albicans* remains as high as 40–70%, especially in severely immune deficient patients.^16^ No approved vaccine is available yet to mitigate any fungal infections.^17^ Therefore, the identification of new drug targets and potential vaccine candidates are of paramount importance. Genomic diversity in the form of base substitutions, indels, ploidy variation, karyotypic changes, and loss of heterozygosity (LOH) is frequently observed in *C. albicans*.^18^ While genomic alteration is deleterious to cells, in certain contexts, it facilitates rapid adaptation to unfavorable environments and is often considered a hallmark of pathogenicity, drug resistance, and cancer development.^19,20^ The opportunistic fungal pathogen *C. albicans* has emerged as an ideal model organism to study the association of genome dynamics with its pathogenesis. Since Polδ takes part in several DNA transaction processes, it might play a key role in the genome stability of *C. albicans*. Therefore, this study was designed to gain insights into the role of Polδ, especially its non-essential subunit Pol32 in genome stability and *C. albicans* biology. We find that CaPol32 plays a critical role in Polδ’s function, and its absence results in complete attenuation of fungal pathogenesis.

## Results

### Identification of the Pol32 subunit of CaPolδ and its interaction with PCNA

**Figure 1.**
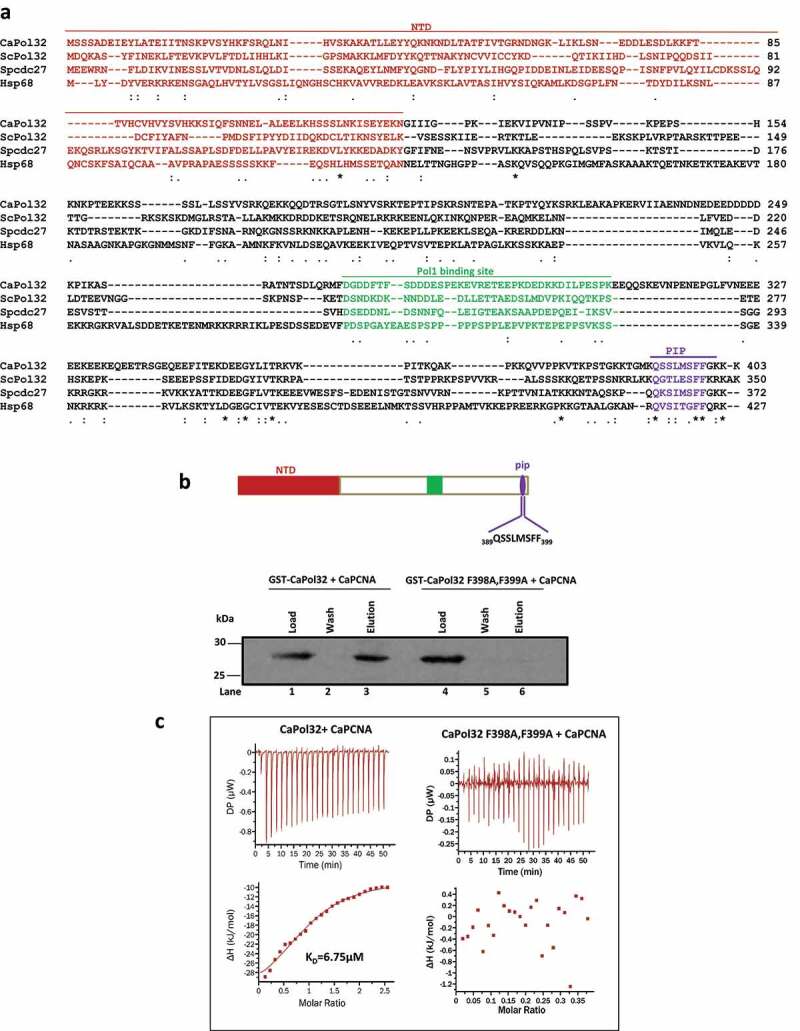
*In silico* analyses and identification of PIP motif of CaPol32. a. Multiple amino acid sequence alignment of CaPol32 with ScPol32, SpCdc27, and human p68 (Hsp68) using CLUSTAL W. Identical residues are designated by * and similar residues are designated with symbols: . N-terminal domain (NTD), Pol1 binding site, and PIP motif are shaded with brown color, green, and violet color, respectively. b. Glutathione sepharose beads bound with GST-CaPol32 (Lanes 1–3) and GST-CaPol32 (F398A, F399A) (Lanes 4–6) were mixed with CaPCNA in the equilibration buffer. After the incubation, beads were washed with equilibration buffer and bound PCNA was eluted by protein loading dye. Various fractions were resolved in 12.5% SDS-PAGE and binding of PCNA was detected by an anti-CaPCNA antibody. c. ITC analysis of binding of CaPCNA with CaPol32 and CaPol32 (F398A, F399A). In each figure, the upper panels show the measure of heat exchanges during each CaPCNA injection, while the lower panels show enthalpy changes as a function of the molar ratio of CaPol32 or CaPol32 (F398A, F399A) binding to a CaPCNA monomer.

To identify the putative homologue of *S. cerevisiae* Pol32 in *C. albicans*, we performed a BLAST search in its genome database (http://www.candidagenome.org/) and found orf19.2465 as an uncharacterized protein that could encode for the smallest subunit of Polδ. The putative *CaPOL32* gene (C1_05850W_A/B) is located in the largest chromosome Chr 1 and both the alleles display synonymous substitution suggesting the expression of a single protein with exactly similar amino acid composition (Length = 403 aa; MW = 46.0 kDa). Our primary sequence alignment of CaPol32 with already characterized Pol32 homologues showed a mere 17–25% identity (Figure 1a). Pol32 from *S. cerevisiae* is known to possess mutually exclusive-binding sites such as the N-terminal 103 aa (NTD), middle 269–310 aa, and C-terminal PIP for Pol31, Pol1, and PCNA, respectively. Except for the canonical PIP motif, the other two binding regions are relatively poorly conserved in CaPol32. Since CaPol32 showed poor conservation of amino acids, we sought to compare its structure with ScPol32. ScPol32 (PDB:6P1H) was taken as a template and the corresponding model structure of CaPol32 was predicted. Similarly, the model structures of the PIP motif of Pol32 from *C. albicans, S. cerevisiae*, and *S. pombe* were predicted using the PEP fold 3.0 online server. Despite the limited amino acid similarity, NTD from Sc- and Ca-Pol32 displayed exceptionally overlapping V-shaped structures and formed a winged helix-turn-helix (wHTH) domain (Supplementary Figure 1i and ii). Similarly, the PIP structures of ScPol32, p68, and CaPol32 were indistinguishable and they formed a 3_10_ helix that stably bound to the hydrophobic pocket of PCNA (Supplementary Figure 1 iii and iv). To authenticate the involvement of the PIP motif of CaPol32 in PCNA binding, the highly conserved aromatic amino acids (F398 and F399) of the putative PIP motif were mutated to alanines as these residues in ScPol32 are involved in hydrophobic interaction with the inter-domain connecting loop of PCNA.^21^ The wild-type and site-directed CaPol32 (F398A, F399A) mutant proteins were purified, and their interaction with CaPCNA was monitored by GST-pulldown and Isothermal calorimetry (ITC) analyses. An equimolar concentration of CaPCNA was incubated with wild-type or mutant GST-CaPol32 and a pull-down assay was performed using glutathione-Sepharose beads (Figure 1b). While the wild-type GST-Pol32 was able to pull down CaPCNA (compare lanes 1 and 3), GST-CaPol32 F398A, F399A protein failed to interact with CaPCNA and thus, no PCNA was precipitated in the elution fraction (compare lanes 4 and 6). For the ITC assay, CaPCNA was injected into the sample cell of the calorimeter either containing wild type or mutant of CaPol32, and heat exchange was monitored (Figure 1c). While the titration of CaPCNA against the wild-type protein resulted in an exothermic reaction with the estimated values of ΔH, ΔG, and K_D_ as ~-27 kcal/mol, ~-29.5 kcal/mol, and ~6.75 μM, respectively, no perceivable heat change was detected when PCNA was titrated against the PIP mutant of CaPol32 or the buffer alone. The titration assay also revealed that one molecule of CaPol32 binds to one molecule of a trimeric PCNA. These results suggested that like in ScPol32 and human p68, CaPol32 also possesses a bonafide PIP motif (392-QSSLMSFF-399) at the extreme C-terminal end.

### PCNA interaction by Pol32 is indispensable for Polδ’s function in *C.* *albicans*

*S. cerevisiae* cells harboring a mutant Pol32 that is defective in PCNA binding did not show any noticeable growth defects in cold temperature and in the presence of hydroxyurea (HU).^3,7^ We examined the phenotypes of CaPol32 and its PIP motif by expressing them at the original genomic locus of a *pol32* homozygous deletion strain (*pol32*ΔΔ) of *C. albicans* (**Supplementary Figures 2 and** Figure 2). Surprisingly, *C. albicans* cells possessing CaPol32 with F398A, F399A mutation exhibited almost similar sensitivity to HU, UV radiation, and high temperatures (42°C) as shown by the *pol32* null strain (Figure 2a). At 42°C or 32–48 J/m^2^ of UV exposure or in the presence of 20–30 mM HU, both wild type and *pol32*ΔΔ::*POL32* strains grew normally; however, almost no growth was observed in the cells expressing CaPol32 PIP mutant. Contrarily and as reported earlier also, *S. cerevisiae pol32*Δ cells possessing PIP mutant grew as good as the cells expressing the wild-type Pol32 at all temperatures tested and upon HU and UV radiation exposures. However, *pol32*Δ *S. cerevisiae* cells exhibited slow growth phenotypes at 16° and 37°C and sensitivity to HU and UV. To further strengthen this result, colony forming units (CFU) of each strain were determined in the presence of genotoxic agents and found that while in *C. albicans*, the PIP motif of Pol32 appears to be as critical as the full-length protein, in *S. cerevisiae*, Pol32 subunit possesses other essential functions that can be separated from the PCNA interaction (Figure 2b). To find out whether the essential role of the PIP motif of CaPol32 is also required during DNA repair synthesis by Polδ, *C. albicans* and *S. cerevisiae* cells expressing wild type or Pol32 PIP mutant were subjected to MMS, TBHP, and cisplatin DNA damaging agents. Surprisingly, both the wild type and PIP mutant of Pol32 conferred protection to genomic DNA from damage as only the *pol32* defective strains of *C. albicans* and *S. cerevisiae* showed susceptibility to various DNA damaging agents (Figure 2c). This result suggested that in genomic DNA replication, PCNA interaction by Pol32 subunit of CaPolδ is indispensable, whereas for most of the DNA repair function PCNA binding to CaPol32 may not be essential, except when cells are exposed to UV radiation.

**Figure 2.**
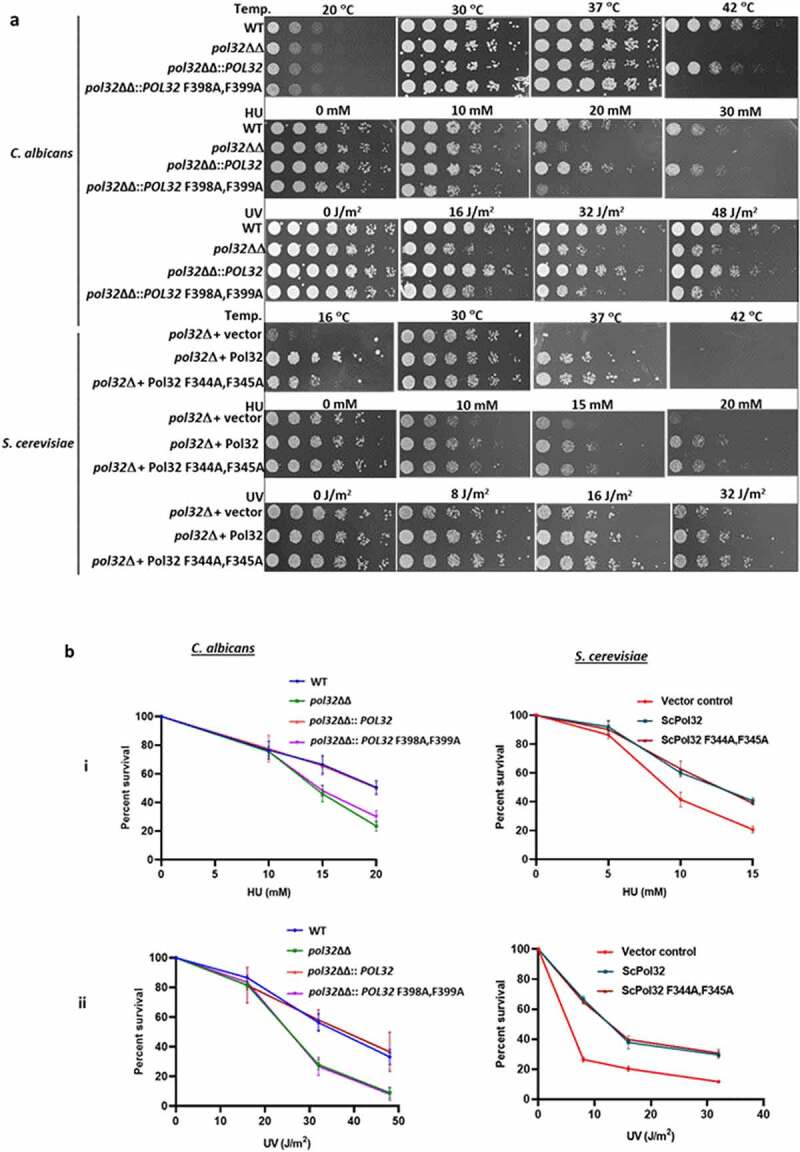
PIP motif of CaPol32 is essential for CaPolδ’s function. a. Serially diluted cells of WT, *pol32*ΔΔ, *pol32*ΔΔ::*POL32*, *pol32*∆∆::*POL32* F398A, F399A C. albic*ans* strains and *pol32Δ* deletion *S. cerevisiae* strain harboring vector alone or ScPol32 or ScPol32 F344A, F345A were spotted on YPD plates with the indicated concentration of HU. Similar YPD plates spotted with various strains were exposed to different doses of UV. All the plates were incubated at 30°C for 48 hr and then imaged. For the temperature sensitivity, YPD plates were incubated at 16°C, 30°C. 37°C, and 42°C for 48 hr and then photographed. b. CFU analysis of various strains of *C. albicans* and *S. cerevisiae* was performed by diluting logarithmically growing cells to 400 cells/ml and from which 250 µl cells were spreaded on YPD plates with HU (i) and UV treatments (ii). c. Equal number of cells of WT, *pol32*ΔΔ, *pol32*ΔΔ::*POL32*, *pol32*∆∆::*POL32* F398A, F399A C. albica*ns* and *pol32*Δ S. cerevisiae strain harboring a vector alone or ScPol32 or ScPol32 F344A, F345A plasmids were spotted on YPD plates without or with MMS, cisplatin, and TBHP. All the plates were incubated at 30°C for 48 hr and then imaged.

**Figure 2.**
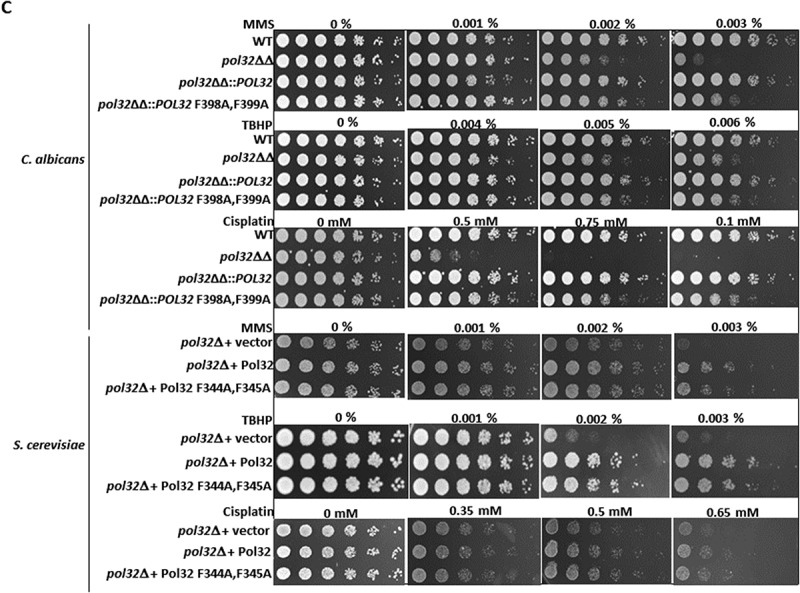
(Continued).

### Loss of PCNA interaction by the Pol32 subunit of CaPolδ renders chromosomal instability

**Figure 3.**
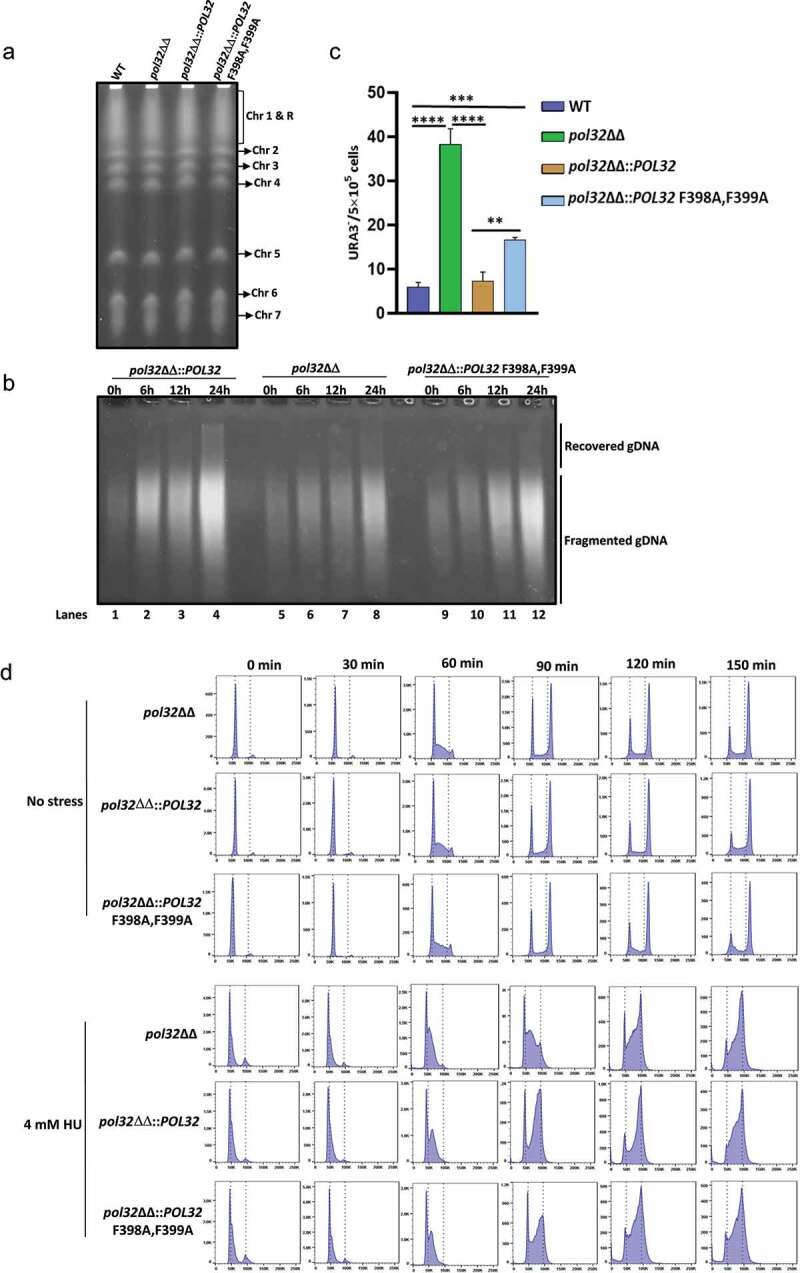
Role of CaPol32 in chromosomal stability and cell cycle progression. a. DNA plugs were prepared for various strains of *C. albicans* and resolved by PFGE. b. Alkaline agarose gel electrophoresis of total DNA isolated from *Pol32*ΔΔ::*POL32* (lanes 1–4), *pol32*ΔΔ (lanes 5–8), and *pol32*ΔΔ::*POL32* F398A, F399A (lanes 9–12) strains of *C. albicans* during the recovery period after HU treatment was carried out. After neutralization, DNA fragments were visualized by ethidium bromide staining. c. The efficiency of LOH of various strains of *C. albicans* was measured by estimating the number of FOA resistant colonies. d. Synchronized cells were chased for 150 mins at an interval of 30 mins to check the cell cycle progression of *C. albicans* cells without or with HU treatment. Cells were stained with cytox green and a single cell gated population was analyzed by flow cytometry.

In order to investigate the occurrence of genomic alterations that could have happened due to the loss of the PCNA interaction motif of Pol32 or due to the complete deletion of the *POL32* gene, we compared the karyotype of *C. albicans* strains (Figure 3a). Our pulse field gel electrophoresis (PFGE) analysis of total genomic DNA revealed no gross chromosomal rearrangement in any of the strains (WT, *pol32*ΔΔ, *pol32*ΔΔ::*POL32, pol32*ΔΔ::*POL32* F398A, F399A), and DNA from all the eight chromosomes migrated according to their molecular sizes as of the parental *C. albicans* strain. Thus, despite the absence of Pol32, the overall structure and number of chromosomes in these cells were not greatly affected. To understand the role of Pol32 in processive DNA synthesis by CaPolδ, these strains were exposed to a sub-lethal dose of HU and further allowed to replicate DNA by growing them on fresh media. Since HU depletes cellular dNTPs level, a low processive DNA polymerase becomes inefficient in completing timely replication of the genome, therefore single-stranded DNA and breaks on DNA often get accumulated. Thus, the accumulation of fragmented smaller sized chromosomal DNA is indicative of compromised processive DNA synthesis. Alkali agarose gel electrophoresis of total genomic DNA isolated at various time points of recovery revealed that an equal amount of genomic DNA degradation was found in each cell type prior to recovery (Figure 3b**, lanes 1, 5, and 9**). With the increase in the duration of the recovery period, a comparatively higher accumulation of larger DNA fragments was observed in WT than that in *pol32*ΔΔ or the cells expressing Pol32 PIP mutant (lanes 2–4, 6–8, and 10–12). Since F398A, F399A mutations in Pol32 abrogated binding with PCNA, the accumulation of fragmented DNA in *C. albicans* strain expressing Pol32 PIP mutant even after 24 hr of recovery phase suggested a critical role of PCNA interaction of Pol32 in Polδ’s function in the cell. Next, we investigated the efficiency of spontaneous LOH events in *C. albicans* cells using a counter-selectable *URA3* gene marker. Heterozygosity of *URA3* was artificially created in different genetic backgrounds of *C. albicans* by knocking out one of the alleles and such strains were subjected to 5FOA treatment to measure LOH frequency. Cells grow in the presence of 5FOA, only when the other *URA3* copy becomes nonfunctional. The appearance of 5FOA^R^ colonies suggests an increased rate of LOH in that corresponding strain of *C. albicans* (Figure 3c). The efficiency of LOH was negligible in the wild type *C. albicans* (6–9 colonies per 5 × 10^5^ cells), whereas it increased by 8–10 folds and 2–4 folds in the *POL32* deficient and strain possessing PIP mutant of Pol32, respectively. Interestingly, a single copy integration of the *POL32* gene into the *pol32ΔΔ* strain exhibited a similar level of LOH frequency as by a diploid WT strain, thus the gene dosage of *POL32* in regulating LOH frequency appears not to be critical in *C. albicans*.

*S. cerevisiae* cells lacking Pol32 exhibit a delay at the G2/M phase of the cell cycle and accumulate non-segregated chromosomes.^22^ To decipher a possible similar role of CaPol32, synchronized populations of *pol32*ΔΔ and its derivative strains *C. albicans* cells at the G1 phase of the cell cycle were treated with or without HU, and the cell cycle progression of the gated single nucleated cells was monitored at different time intervals by flow cytometry analysis as described previously.^23^ Without HU, all three strains (*pol32*ΔΔ, *pol32*ΔΔ::*POL32*, and *pol32*ΔΔ::*POL32* F398A, F399A strains followed a normal cell cycle and took ~90 min to complete a cycle from 2 N (G1) to 4 N (G2/M) (Figure 3d). Up to 150 mins, the progression profiles of all of the cells remained undistinguishable suggesting that the loss of the *POL32* gene or PIP motif mutant of *POL32* did not result in any replication stress to activate cell cycle arrest under optimal growth conditions. However, chronic exposure to a nontoxic dose of HU (4 mM) was sufficient enough to delay the cell cycle progression and more cells were accumulated at the S phase of *pol32*ΔΔ and *pol32*ΔΔ::*POL32* F398A, F399A *C. albicans* cells. Altogether, these results suggested that although Pol32 is non-essential for growth, its binding to the processivity factor PCNA stimulates processive DNA synthesis by Polδ, and its absence leads to a higher rate of spontaneous and HU induced chromosomal instability, and early to late S phase arrest.

### Genomics of *pol32*ΔΔ and its isogenic parental strain of*C.* *albicans*

Genomic analysis of clinical isolates of *C. albicans* and isolates subjected to environmental stresses revealed that many of those contained loss of heterozygosity (LOH) and large-scale genomic changes such as whole or segmental chromosome aneuploidy.^24,25^ Although our PFGE analysis of *pol32*ΔΔ *C. albicans* chromosomal DNA did not show any abnormal chromosomal mobility, the *POL32* defective strain exhibited an increased rate of LOH. To further strengthen our understanding of the role of Pol32 in genome stability, whole-genome sequencing of the *pol32*ΔΔ and its parental wild-type strain of *C. albicans* was performed concurrently and compared with the reference genome. Since mutations have been identified in *C. albicans* isolates passaged both *in vitro* and *in vivo* conditions during laboratory culture and in the mammalian host by earlier reports,^25,26^ it was important to re-sequence our laboratory parental wild-type strain. *C. albicans* genome consists of eight chromosomes, with the reference isolate SC5314 harboring ∼70,000 heterozygous positions representing ∼0.5% of the 14.3 Mb genome. After filtering, trimming and removal of redundans from the raw reads, a total of 259 and 234 contigs corresponding to 14528623 and 14626883 bp of total reads were obtained for wild type and *pol32*ΔΔ genome, respectively, and are equivalent to the reference genome size. First, we used a web-based tool YMAP (Yeast Mapping Analysis Pipeline) that generates a chromosomal map and analyzes the genome sequence to map copy number variations (CNV), SNPs, and LOH.^27^ This pipeline uses the FASTQ files, makes stringent corrections, and compares with the reference sequence to result in a HapMap that illustrates each chromosome (Figure 4a i and II, and Supplementary Figure 3). The constriction that divides each chromosomal arm denotes a centromere. The X-axis represents the chromosomal distance, with 0.2 representing 200 KB. A graph on the left of the chromosome represents the allelic ratio. The right-hand side number represents the ploidy of that specific chromosome, i.e. 2 for two alleles, 1 for a single allele, and any numbers between >1 and <2 represent cases of aneuploidy. The regions with copy number variations are depicted as prominent black histograms along the length of the chromosome. The dots on the X-axis denote the positions of the major repeat sequences (MRS). YMAP distinguishes between the haplotypes of each chromosome (A or B) by using a color code. Heterozygous (AB) single nucleotide polymorphisms (SNPs) are shown as vertical gray bars in the background of each chromosome, and increasing shades of dark gray indicate regions with higher numbers of SNPs. Homozygous SNPs are displayed in cyan for haplotype A and in magenta for haplotype B. Aneuploidy is determined by the weighted average of the colors assigned to the individual SNPs. As shown in Figure 4a, there are two copies of all chromosomes in both the *C. albicans* strains. A large region on the small arm of chromosome 5 (colored in cyan) and a small region close to the MRS on chromosome 2 (colored in magenta) have undergone loss of heterozygosity (LOH). Since these LOH events are commonly found in both strains, these events happened at an early stage and not during passaging in our laboratory (Figure 4a i **and II**). Among these chromosomes, Chr 3 which is relatively smaller in size looks very stable as it has minimal LOH events. The appearance of several new cyan and magenta lines in the *pol32*ΔΔ strain is indicative of loss and gain of homozygosity accumulated due to faulty DNA replication that was more evident in Chr 7. Interestingly, Chr 7 also exhibited localized segmental aneuploidy in the larger arm (chromosome number is estimated to lie between 1.2 and 2). These genetic variations can cause the loss or gain of new phenotypes in the *pol32*ΔΔ strain.

**Figure 4.**
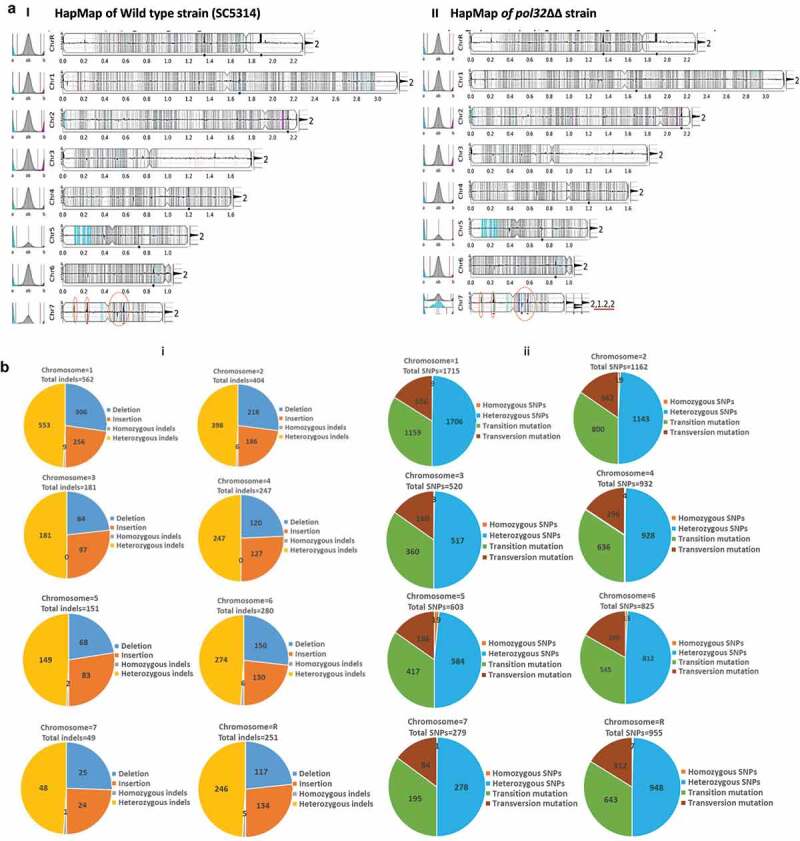
WGS of *pol32*ΔΔ strain of *C. albicans*. a. The raw FASTQ genome sequence files of our laboratory WT (i) and *pol32*ΔΔ (II) strains of *C. albicans* were mapped with reference sequence using Y_MAP_. Y_MAP_ provided the chromosomal map. The gray color lines indicate heterozygous regions in both haplotypes and the cyan and magenta lines indicate heterozygous regions in any of the haplotypes. The histogram at the left arm of the chromosome represents the proportion of each haplotype (assigned as a or b). The histogram at the right end of the chromosome indicates the copy number variation. Chromosome 7 of *pol32*ΔΔ strain showed segmental aneuploidy and brown circles indicate some of the unique variation regions. b. Numbers of Indels (i) and SNPs (ii) specifically accumulated in the genome of *pol32*ΔΔ strain of *C. albicans*. Blue color indicates deletion, brown color indicates insertion, gray color indicates homozygous Indels, yellow color indicates heterozygous Indels, Orange color indicates homozygous SNPs, sky blue color indicates heterozygous SNPs, green color indicates transition mutation, and maroon color indicates transversion mutation. c. Accumulation of Indels and SNPs associated without or with homo/hetero-polymeric repeat regions of *pol32*ΔΔ genome of *C*. *albicans*. d. The frequency distribution of length of deletion and insertion specific to *pol32*ΔΔ genome of *C. albicans.*

**Figure 4.**
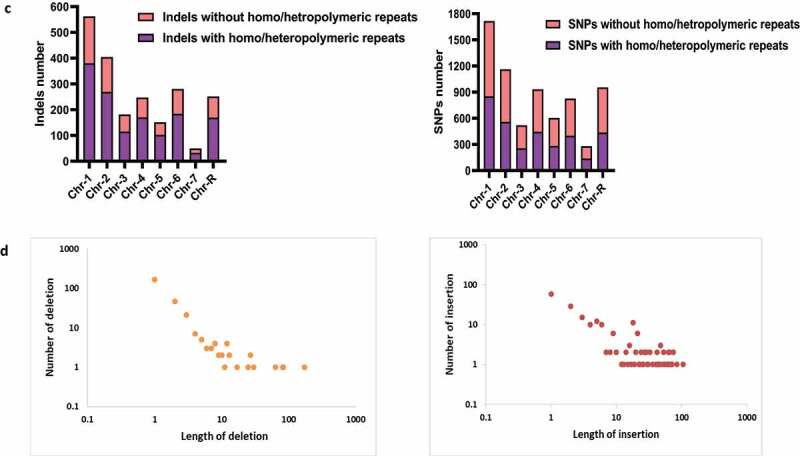
(Continued).

Over a period of time, our laboratory WT SC5314 strain accumulated 7942 indels (insertion + deletion) and 62995 SNPs when we compared it with the publicly available reference sequence (ASM18296v3). A detailed chromosome-wise accumulation of various genetic changes has been illustrated (Supplementary Figure 4 and Supplementary Tables 1 and 2). In addition to these genomic variations, 2125 indels and 6991 SNPs events occurred in the *pol32ΔΔ* genome which might have commenced most likely due to replication stress linked specifically to Pol32 function. This result is also supported by the fact that the loss of Pol32 enhanced 5FOA^R^ LOH efficiency as shown in Figure 3c. A chromosome-wise accumulation of indels and SNPs specific to the *pol32*ΔΔ genome has been depicted (Figure 4b **and** Table 1). Among the chromosomes, as found in the YMAP pipeline Chr 3 accumulated the least number of indels and SNPs in both strains. About 181 indels and 520 SNPs, and 489 indels and 4311 SNPs were accumulated in Chr 3 of *pol32*ΔΔ and WT strain, respectively. Since Chr 3 has no major repeat sequence (MRS) region, the replication slippage events could be a bare minimum in this chromosome and thus, it is fairly stable. Contrarily, Chr 4 and Chr 6 despite being relatively smaller chromosome sizes (~1-1.6 MB) possessed a higher percentage of genetic variations. When we estimated the allelic variations, the WT strain had accumulated 179 homozygous indels, 7763 heterozygous indels, 366 homozygous SNPs, and 62629 heterozygous SNPs. Despite so much accumulation of genomic changes, these changes have not impacted the function of the WT genome as the WT strain has retained its phenotypes and virulence attributes as shown by the referred SC5314 strain. However, in the *POL32* defective genome, 29 homozygous indels, 2098 heterozygous indels, 75 homozygous SNPs, and 6916 heterozygous SNPs additional variations were found that could influence the phenotypes of *C. albicans*.

**Table 1.** Accumulation of indels and SNPs only due to the loss of *POL32* gene in *C. albicans* strain.

Chr. no	Total indels	Deletion	Homozygous deletion	Frameshift variants	Heterozygous deletion	Insertion	Homozygous insertion	Frameshift variant	Heterozygous insertion
1	562	306	5	0	301	256	4	1	252
2	404	218	3	1	215	186	3	0	183
3	181	84	0	0	84	97	0	0	97
4	247	120	0	0	120	127	0	0	127
5	151	68	1	0	67	83	1	0	82
6	280	150	4	3	146	130	2	0	128
7	49	25	0	0	25	24	1	0	23
R	251	117	2	1	115	134	3	0	131
total	2125	1088	15	5	1073	1037	14	1	1023
Chr. No	Total SNPs	Homozygous SNPs	Transition mutation	Transversion mutation	Heterozygous SNPs	Transition mutation	Transversion mutation	Total transition mutation	Total transversion mutation
1	1715	9	6	3	1706	1153	553	1159	556
2	1162	19	16	3	1143	784	359	800	362
3	520	3	0	3	517	360	157	360	160
4	932	4	2	2	928	634	294	636	296
5	603	19	9	10	584	408	176	417	186
6	825	13	5	8	812	540	272	545	280
7	279	1	1	0	278	194	84	195	84
R	955	7	5	2	948	638	310	643	312
Total	6991	75	44	31	6916	4711	2205	4755	2236

### Fidelity and replication slippage associated with higher mutation rate in *pol32ΔΔ* strain

A mutant of Polδ that is defective in proofreading activity (L612M) frequently introduces AT to GC transitions with a preference for T•dG mispairs and CG to TA transition through G•dT mispairs.^28^ Another class of Pol δ-specific mutations consists of GC to TA transversions formed mainly by C•dT rather than G•dA mispairs.^29^ We observed ~2 folds more transition than transversion mutational signature in *pol32*ΔΔ *C. albicans* (Table 1). Out of 75 homozygous SNPs, 44 were transition and 31 were transversion mutations. Similarly, out of 6916 heterozygous SNPs, 4711 were transitions and 2205 transversions. Chromosomal features are known to govern mutational patterns in eukaryotic organisms. Repetitive regions, sub-telomeric regions, and gene families encoding cell surface proteins involved in host adhesion are the hotspots in the *C. albicans* genome where the mutation spectrum alters frequently.^25^ Our NGS analyses revealed that about 67% of variations (1421 out of total 2125 indels) were seen in homo- or hetero-polymeric repeats regions of the chromosomes (Figure 4c, Table 2). These repeats vary from 1 to 17 nucleotides of random combinations and are located across the chromosomes. However, the accumulation of SNPs was repeat-regions independent as an about equal percentage of SNPs were observed in both polymeric repeats (3364) and without any repeat regions (3627). This suggests that more than the fidelity, it is the processivity of Polδ that gets affected in the absence of Pol32 and contributed to high indel rates, probably due to the expansion or contraction of repeat sequences. Out of 15 homozygous deletions and 14 homozygous insertions, only 5 deletions and 1 insertion were frameshift mutations located in the uncharacterized protein encoding genes (Table 1). These deletions and insertions vary from 1 to ~100 bp, and the large-sized deletions were less frequently accumulated than the large-sized insertions (Figure 4d).

**Table 2.** Repeat regions specific accumulation of indels and SNPs only due to the loss of *POL32* gene in *C. albicans* strain.

Chr. no	SNPs with homo/heteropolymeic repeats	Homozygous SNPs	Heterozygous SNPs	SNPs without homo/heteropolymeric repeats	Homozygous SNPs	Heterozygous SNPs
**1**	851	3	848	864	6	858
**2**	558	11	547	604	8	596
**3**	255	3	252	265	0	265
**4**	444	3	441	488	1	487
**5**	283	7	276	320	12	308
**6**	400	8	392	425	5	420
**7**	137	1	136	142	0	142
R	436	5	431	519	2	517
Total	3364	41	3323	3627	34	3593
Chr. no	Indels with homo/hetropolymeic repeats	Homozygous indels	Heterozygous indels	Indels without homo/hetero polymeric repeats	Homozygous indels	Heterozygous indels
1	380	7	373	182	2	180
2	269	1	268	135	5	130
3	115	0	115	66	0	66
4	170	0	170	77	0	77
5	102	1	101	49	1	48
6	184	4	180	96	2	94
7	32	1	31	17	0	17
R	169	2	167	82	3	79
Total	1421	16	1405	704	13	691

### Pol32 regulates morphological switching in *C.* *albicans*

**Figure 5.**
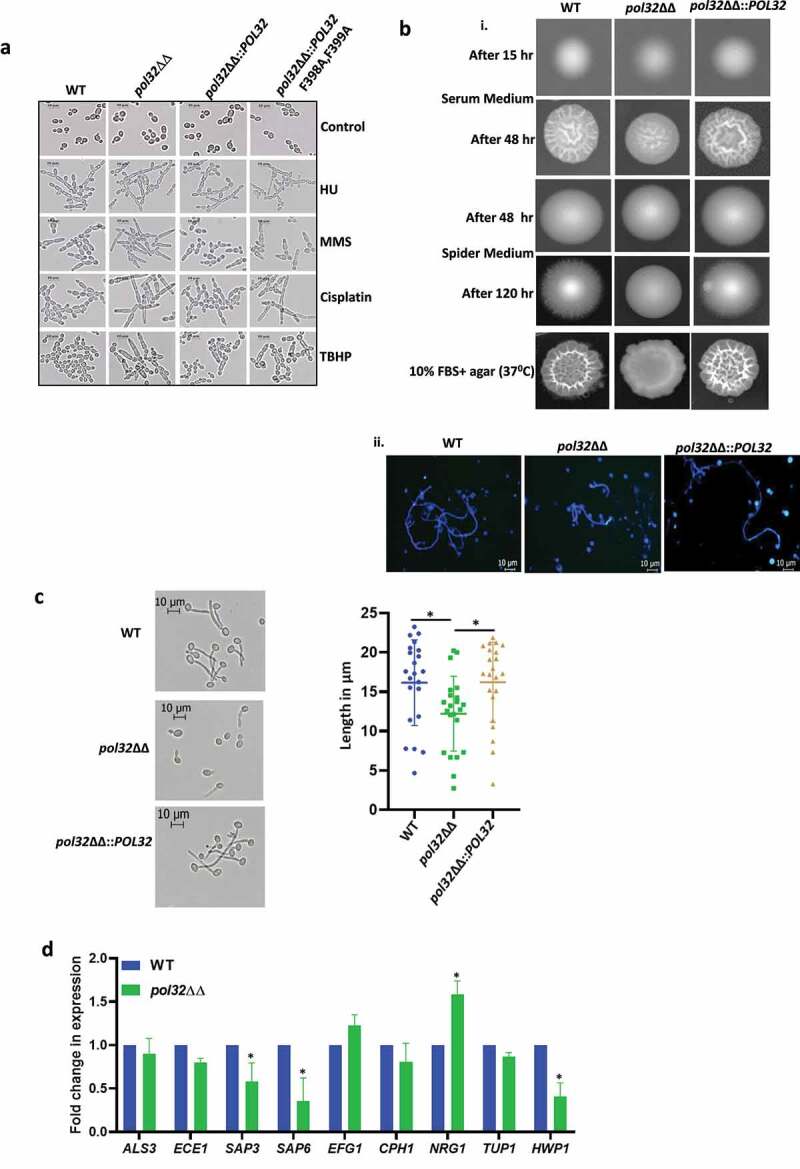
Morphogenesis of *C. albicans* strains. a. Cells from various strains of *C. albicans* were grown in liquid media for 4 hr in the presence or absence of various genotoxic agents as indicated and morphology was observed under a light microscope. b. Overnight cultures of various strains of *C. albicans* were diluted to 500 cells/ml, from which 200 µl cells were spreaded on YPD plates with 10% FBS, spider agar plates, and 10% FBS agar plates. All the plates were incubated at 37°C and images were taken at mentioned time points (i). Cells from YPD +10% FBS agar plates were taken, fixed, and stained with CFW, and images were captured under a fluorescent microscope (ii). c. Cells of WT, *pol32*ΔΔ and *pol32*ΔΔ::*POL32* strains were grown for 1 hr in YPD liquid media containing 10% FBS and observed under the light microscope. The length of the germ tubes was measured using image j software and represented in dot plots. d. Expression of various virulence and hyphal associated genes were quantified by real-time PCR. *GAPDH* was used as a control. The results are presented as mean ± standard deviation. Asterisks indicate the statistically significant differences compared to the results of the WT using the student t-test (*P < .05).

Under standard growth conditions, WT *C. albicans* cells are round-shaped. However, inadequate cellular dNTPs concentration and accumulation of DNA lesions inflict genomic stress that gets translated into filamentous growth in *C. albicans*.^30,31^ DNA replication defects, delay in cell cycle progression, and activation of checkpoints also regulate filamentation in *C. albicans*.^32^ Since the deletion of *POL32* caused genome instability and a significant delay in cell cycle progression under HU treatment, we investigated the association of CaPol32 function with morphological transition. *C. albicans* cells were subjected to susceptible but sub-lethal doses of HU, MMS, cisplatin, and TBHP in liquid cultures for 4 h, and their morphology was examined (Figure 5a). In the presence of genotoxic agents, cells of all the strains of *C*. albicans (WT, *pol32*ΔΔ, *pol32*ΔΔ::*POL32*, and *pol32*ΔΔ::*POL32* F398A, F399A) switched their morphology to pseudohyphae, and only a very low percentage (<10%) of cells were either isolated or chained morphology. This result implicated that the loss of *POL32* has no major effect on the genotoxins induced morphological switching of *C. albicans*. Further, we inspected colony morphology in spider and serum-containing solid media which are also known inducers of filamentation but do not inflict genomic stress.^23^ On the serum-containing media, the colony of *C. albicans* appears rough and wrinkled; while on spider media prominent hairy outgrowths appear on the outer layer of the colony. We observed that at early time points, colonies of both Pol32 proficient and deficient strains looked very similar on both the media, however after prolonged incubation lesser wrinkled and hairy outgrowths colonies were found in *pol32*ΔΔ *C. albicans* cells than the *POL32* proficient (WT and *pol32*ΔΔ::*POL32*) cells (Figure 5b i). The wrinkleless of the colony was directly proportional to filamentation and the CFW staining of the colony from serum containing media substantiated reduced filamentation of *pol32*ΔΔ cells (Figure 5b **ii**). In liquid media, supplementation of 10% serum to YPD leads to germ tube development in WT *C. albicans*.^33^ In comparison to WT and *pol32*ΔΔ::*POL32* cells, the Pol32 deficient cells produced stunted germ tubes (Figure 5c). All these results suggest that Pol32 could play an important role in genomic stress-independent morphology switching.

### Altered expression of hyphal and virulence genes in *POL32* deficient strain of *C.* *albicans*

The morphological transition of *C. albicans* from round to filamentous shape is due to or renders altered expression of several genes such as transcription factors involved in filamentation, agglutinin-like sequence (Als) required for cell-surface adhesion, secreted aspartyl proteases (Sap), and Ece1 for membrane penetration, etc.^34–36^ While *EFG1* and *CPH1* are the transcriptional activators, *NRG1* and *TUP1* are the transcriptional repressors of yeast to hyphal transition.^37^ *HWP1* is a downstream hyphal specific virulence gene. *ECE1* encodes a cytolytic peptide toxin candidalysin that damages epithelial membranes, triggers a danger response-signaling pathway, and activates epithelial immunity.^38^ To determine the effect of the absence of *POL32* in virulence gene expression in *C. albicans*, differential expression of the above-mentioned genes was examined by both real-time and semi-quantitative end-point reverse transcription PCRs (Figure 5d**, Supplementary Fig. 5A, and Supplementary Table 3**). In both the assays, our expression analyses revealed that while we could not detect any noticeable difference in the mRNA levels of *ALS3, ECE1, EFG1, CPH1*, and *TUP1* genes, expression of *SAP3, SAP6*, and *HWP1* genes decreased marginally in *pol32ΔΔ* cells. Interestingly, hyper-expression of the *NRG1* gene was noticed in Pol32 deficient cells, which is a well-known suppressor of filamentation.^37^

### Loss of *Pol32* induces azoles drug resistance in *C.* *albicans*

Azoles are the frontline antifungal drugs, however, overuse of these drugs leads to their resistance.^39,40^ Azoles inhibit the C14α demethylation of lanosterol in fungi, which causes the depletion of ergosterol in the membrane. In *C. albicans*, the *ERG11* gene encodes lanosterol demethylase, a target of azoles. The C-5 sterol desaturase gene (*ERG3*) is another essential enzyme required for ergosterol biosynthesis. Mutations in *ERG3* and *ERG11* that result in an amino acid substitution responsible for their non-functionality alter the membrane fluidity and result in drug resistance. *C. albicans* acquires azole resistance via structural alterations of the drug target, overexpression of the target gene products, an enhanced drug expulsion due to the overexpression of drug efflux transporters, and altering the cell wall and membrane architectures. The hyper-activation of TOR signaling is another important mechanism in *C. albicans* to bypass azole toxicity. Additionally, genome instability has been reported to be associated with multidrug resistance in *C. albicans*.^26,41^ Therefore, a correlation between Pol32 mediated genome stability and drug resistance in *C. albicans* was determined (Figure 6a). Interestingly, Pol32 defective cells exhibited resistance to all three azole drugs- fluconazole, miconazole, and ketoconazole when compared to WT *C. albicans* cells. Even in the liquid growth media, we found that the *pol32*ΔΔ strain was resistant to fluconazole and the growth rate was significantly high in comparison to treated *POL32* proficient (WT and *pol32*ΔΔ::*POL32*) cells (Figure 6b). Interestingly, although Amphotericin B also targets ergosterol of fungal plasma membrane, *pol32ΔΔ* strain of *C. albicans* was hypersensitive. Similarly, in treatments with 5-fluorouracil (5FU) and 5-fluorocytosine (5FC) that target nucleotide metabolism and transcription, similar growth inhibition of both Pol32 proficient and deficient strains were observed. Since the re-integration of a single copy of the *POL32* gene restored antimycotic susceptibility level similar to WT, mutation (/s) in the drugs target site or efflux pumps may not be a reason behind acquiring azole drug resistance and hypersensitivity to other antifungals by *pol32*ΔΔ strain. To understand a possible mechanism of azole drug resistance, first, we checked any modifications in the fungal cell wall and membrane fluidity in the *pol32*ΔΔ strain of *C. albicans* by spotting the cells on SDS, CaCl_2_, and CFW, and compared susceptibility with WT. Surprisingly, the growth of the *pol32* null strain was significantly reduced in the presence of mentioned xenobiotics, while the growth of WT and *POL32* revertant was not perturbed indicating certain modifications in the fungal cell wall and (or) membrane architecture (Figure 6c). To make sure that the plasma membrane localized drug transporters are not affected in *POL32* deficient strains, retention ability of Congo red and berberine in the cells of WT, *pol32*ΔΔ, and *pol32*ΔΔ::*POL32* strains of *C. albicans* was determined by spot analysis. Since these reagents are cytotoxic, retention of these will affect growth. Our spot analyses revealed that the growth of these strains was not affected suggesting the efficient functioning of drug efflux pumps. To strengthen our findings, cells were stained with fluorescent CFW, berberine, and Congo red compounds and analyzed by FACS (Figure 6d and supplementary Figure 6). Since CFW stains the chitin of the fungal cell wall, a higher level of mean fluorescence intensity of *pol32*ΔΔ than the Pol32 expressing cells again confirmed altered cell wall composition. Similarly, the equal staining of WT, *pol32*ΔΔ, and *pol32*ΔΔ::*POL32* cells by berberine and Congo red further ensured the efficient functioning of drug transporters and efflux pumps in these strains. Rapamycin inhibits Tor1 function, therefore, we compared the growth of various strains in the presence of rapamycin and found that the *pol32* null strain was equally sensitive to rapamycin as WT and *POL32*-integrated strains. Thus, it ruled out any hyper-activation of Tor1 signaling in the *POL32* deficient strain and its azole resistance is most likely due to the increased LOH events and modifications in the fungal cell wall and membrane structures.

**Figure 6.**
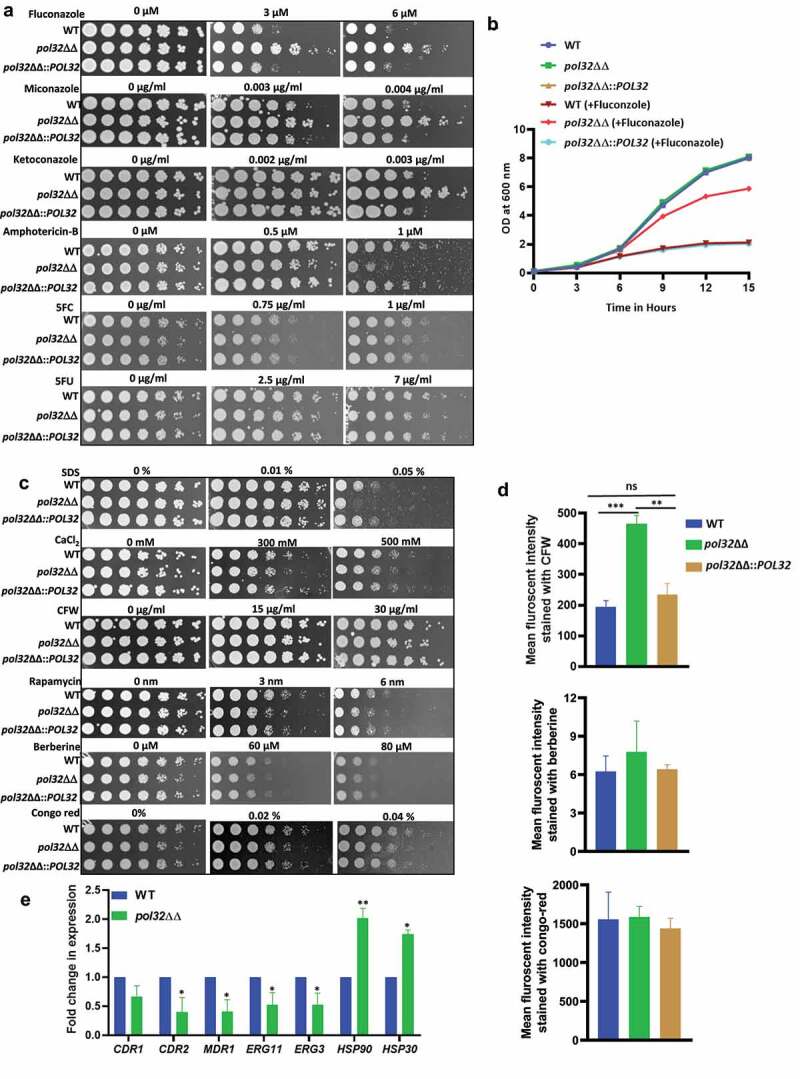
Antifungal drug susceptibility. a. Overnight cultures of WT, *pol32*ΔΔ and *pol32*∆∆::*POL32* strains were serially diluted and spotted on YPD plates without or with the indicated concentration of various antifungal drugs. All the plates were incubated at 30°C for 48 hr and photographed. b. Overnight grown cultures of WT, *pol32*ΔΔ and *pol32*ΔΔ::*POL32* strains were diluted in fresh YPD media and grown at 30°C in the absence or presence of fluconazole (6 μM). The absorbance was measured at OD_600_ for 15 hr at an interval of 3 hr and was plotted using Graph pad prism 8.0. The experiment was performed in biological duplicates and repeated twice. c. Serially diluted cultures of WT, *pol32ΔΔ*, and *pol32*∆∆::*POL32* strains were spotted on YPD plates without or with indicated concentrations of SDS, CaCl_2_, CFW, rapamycin, berberine, and *Congo* red. Plates were incubated at 30°C for 48 hr and photographed. d. Cells from various strains of *C. albicans* were stained with CFW, berberine and *Congo*-red and analyzed by flow cytometry. The mean fluorescence intensity from 3 independent experiments were plotted using Graph Pad Prism 8.0. The results are presented as mean ± standard deviation. Asterisks indicate the statistically significant differences between WT, *pol32*ΔΔ, *pol32*ΔΔ:*:POL32* using the student t test (**P < .01, ***P < .001) e. Quantification of the expression of genes associated with azoles drug resistance by real-time PCR. *GAPDH* was used as a control. The results are presented as mean ± standard deviation. Asterisks indicate the statistically significant differences compared to the results of the WT using the student t-test (*P < .05; **P < .01). f. Serially diluted cultures of WT, *pol32*ΔΔ, and *pol32*ΔΔ::*POL32* were spotted on YPD plates containing either indicated concentration of geldanamycin, trichostatin A, fluconazole, and in combinations. All the plates were incubated at 30°C for 48 hrs and photographed. g. The absorbance of *pol32*ΔΔ strain in YPD media in the presence or absence of fluconazole alone, geldanamycin alone or in a combination of both at 30°C was measured and plotted. h. The absorbance of *pol32*ΔΔ strain in YPD media in the presence or absence of fluconazole alone, trichostatin A alone or a combination of both at 30°C was measured and plotted.

**Figure 6.**
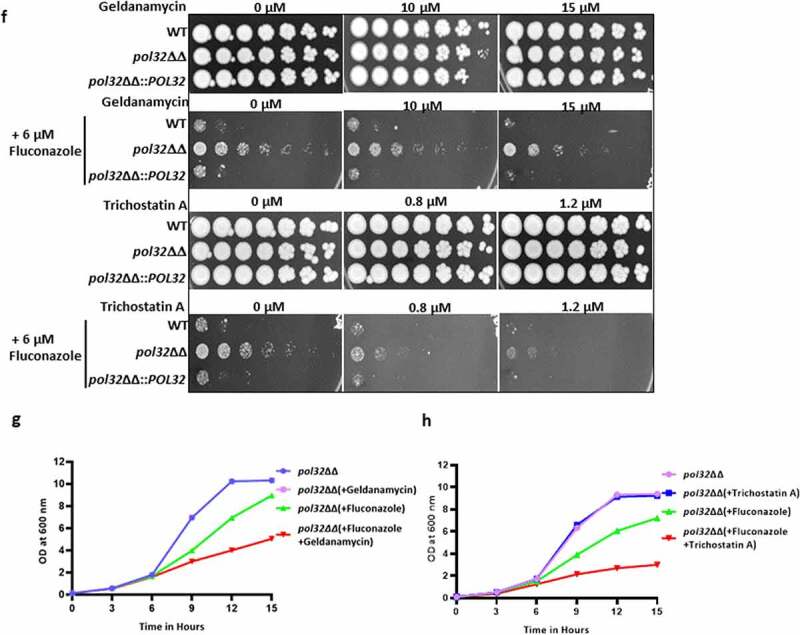
(Continued).

### Heat-shock proteins but not drug transporters are involved in azoles resistance in the Pol32 defective strain of *C.* *albicans*

In addition to the drug efflux transporters (Cdr1, Cdr2, and Mdr1), the involvement of heat shock proteins in the drug resistance of *C. albicans* has been demonstrated.^42^ Hsps are the ubiquitous proteins usually expressed in response to thermal stress; however, studies also show their activation to non-thermal stressors such as e.g., heavy metals and oxidative stress. Hsps in *C. albicans* confer resistance to antifungal drugs by regulating various signaling pathways, such as calcium-calcineurin, MAPK, Ras1-cAMP-PKA, and cell cycle control signaling.^43^ To understand the mechanism of azole resistance of *pol32ΔΔ* strain at the molecular level, the expression of drug efflux pumps and Hsps was determined by using real-time and semi-quantitative RT-PCRs (Figure 6e**, Supplementary Fig. 5B, and Supplementary Table 3**). Our expression analyses revealed that while we could detect a marginal decrease in the mRNA levels of *CDR1, CDR2, MDR1, ERG3*, and *ERG11* genes, expression of *HSP30* and *HSP90* genes increased significantly in *pol32ΔΔ* cells. Overexpression of *CDR1, CDR2, MDR1*, and *ERG11* genes is usually found in drug-resistant clinical isolates. Since we observed lowered expression of these genes in the *pol32*ΔΔ strain of *C. albicans*, it suggested that these genes may not be responsible for azole resistance. However, decreased expression of the *ERG3* gene has been reported to be one of the causative factors of azoles resistance in clinical isolates of *C. albicans* and could be a causative factor in *pol32*ΔΔ strain as well.^44^ Inhibition of Hsp90 function results in azoles sensitivity in *C. albicans* planktonic cells and biofilms.^45,46^ Hsp30 is a plasma membrane heat shock protein, and its role in MDR has not been deciphered yet. Hsp90 inhibitors have been shown to have synergistic effects in combination with fluconazole (FLC) against FLC-resistant *C. albicans*.^47,48^ To confirm that the azoles resistance by *pol32ΔΔ* strain indeed occurred due to the overexpression of Hsp90, we performed spot and growth curve assays in the presence of Hsp90 inhibitors geldanamycin, trichostatin A, and fluconazole alone or a combination of these reagents (figure 6f-h). Geldanamycin binds to the ADP/ATP-binding pocket of Hsp90, whereas trichostatin A blocks the acetylation of Hsp90 which is critical for Hsp90’s role in the emergence of azoles resistance.^42^ Interestingly, in both assays, no growth reduction was observed for the *pol32*ΔΔ strain in the presence of geldanamycin, trichostatin A, and fluconazole alone. However, a significant reduction in the growth rate was observed in the presence of fluconazole with geldanamycin or trichostatin A. Thus, replication stress due to the loss of *POL32* might be one of the reasons behind the hyper-expression of Hsp30 and Hsp90 and a possible cause of azoles resistance in *C. albicans*.

### Ultrastructure of *POL32* defective *C.* *albicans* strain reveals alteration in cell wall composition and thickness

**Figure 7.**
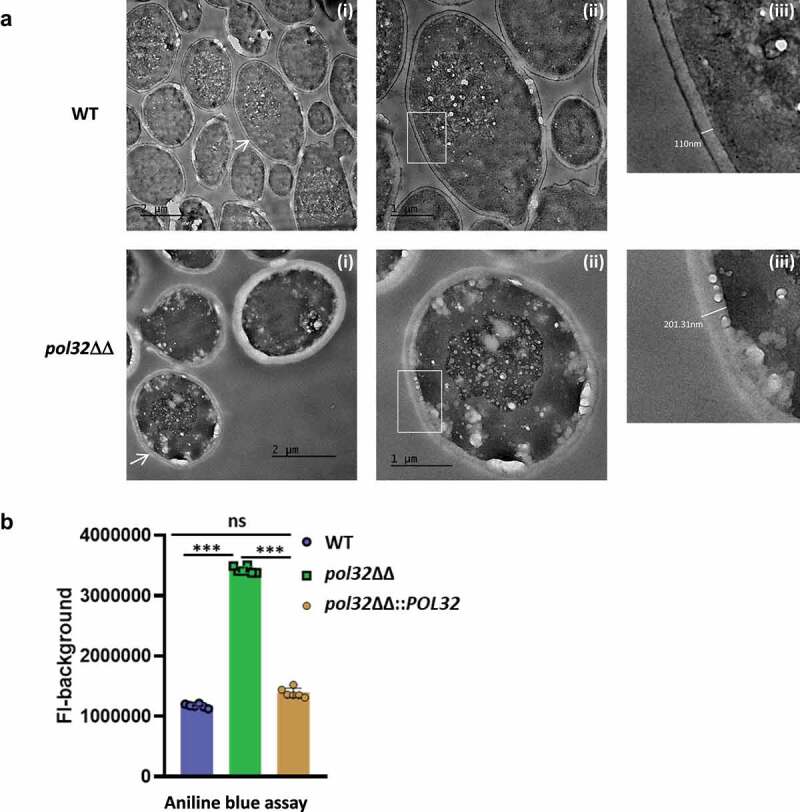
Ultrastructure of *C. albicans* cell. a. TEM images of ultra-thin sections of WT, and *pol32*ΔΔ depicting assembly of intact yeast cells, scale bar = 2 μm (i), an individual cell, scale bar = 1 μm (ii), and (iii) the thickness measurement of individual cell wall. Samples visualized up to a magnification of 14000X. Arrow indicates the zoom in image of the marked cell. b. Glucan content of the WT, *pol32*ΔΔ and *pol32*ΔΔ::*POL32* cells as estimated by aniline blue assay.

The cell wall of *C. albicans* is composed of three layers, the innermost layer is enriched in chitin, the middle layer contains 1–3 and 1–6 β-linked glucans, and the outermost fibrillar layer has mannoproteins. Chitin and glucan layers are well organized to determine the rigidity and shape of the cell wall, while the mannans layer is less structured with low permeability and porosity.^49^ Since the susceptibility and effective staining of *POL32* deficient cells to CFW indicated deformity of the cell wall with more chitin content, to confirm the finding, we determined and compared the ultrastructures of log phase cultured wild type and *pol32*ΔΔ *C. albicans* cells by transmission electron microscopy (Figure 7a). The microscopy of the ultra-thin sections (85 nm) of *C. albicans* cells revealed a differential morphology of both the cells. While the wild-type cells were slightly elongated round shaped, *pol32*ΔΔ cells were perfectly oval shaped. The *pol32*ΔΔ cells exhibited higher cell wall thickness and overall size compared to WT *C. albicans*. We found that the average thickness of the cell wall was 234 nm and 90 nm of *pol32*ΔΔ and WT cells, respectively. The higher thickness of the cell wall of *pol32*ΔΔ cell was due to the bulkiness of the middle glucan layer, and the aniline blue assay confirmed ~ 3-fold more amount of glucan content in the cell of *pol32*ΔΔ than that in WT and *pol32*ΔΔ::*POL32* cells (Figure 7b). Since the cell wall of *C. albicans* acts as an interface between the fungus and host cells, the morphological modification of *POL32* deficient cells could affect its pathogenicity in addition to its role in azoles drug resistance.

### Loss of *POL32* induces biofilm formation of *C.* *albicans*

**Figure 8.**
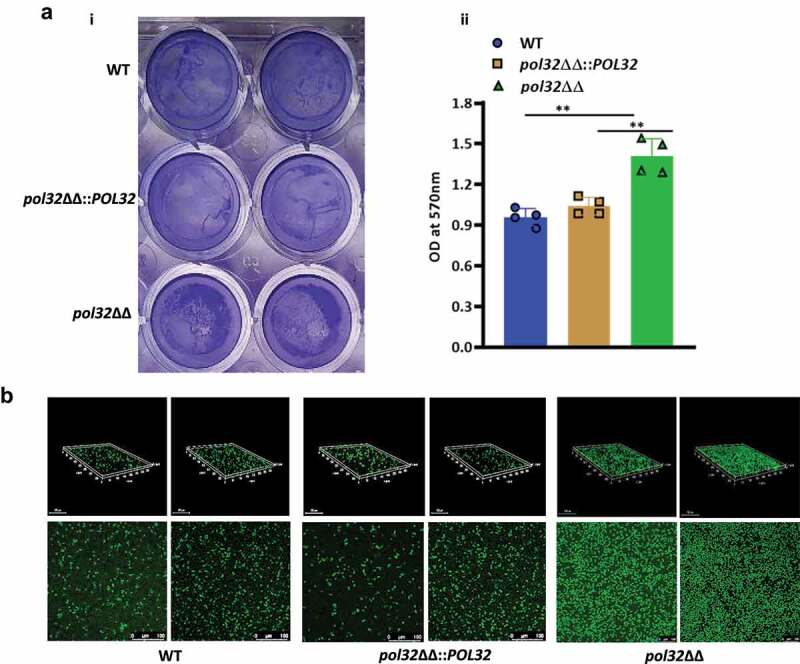
Biofilm estimation by crystal violet assay and Confocal Laser Scanning Microscopy (CLSM). a. The WT, *pol32*ΔΔ and *pol32*ΔΔ::*POL32* strains of *C. albicans* (n = 4) were grown in YPD media with 10% FBS in 24 wells polystyrene plates for 24 hrs at 37°C. The wells were washed with PBS and stained with 0.05% crystal violet (i) and their absorbance was measured at 570 nm (ii). The obtained absorbance was plotted using Graph pad prism 8.0. The results are presented as mean ± standard deviation. Asterisks indicate the statistically significant differences compared to the results of the WT using the student t-test (**P < .01). b. WT, *pol32*ΔΔ and *pol32*ΔΔ::*POL32* strains of *C. albicans* (n = 2) were grown on 6-wells polystyrene plates containing glass coverslips for 24 hrs at 37°C. The biofilm formed on the coverslip was stained with 1% acridine Orange and images were captured using Leica TCS SP8 confocal system with an excitation wavelength of 483 nm and emission wavelength of 510 nm. Upper panel is 3D image and lower panel is 2D image.

Biofilms are the microbial communities embedded in a matrix of extracellular polymers generally attached to a surface and a leading cause of chronic human fungal infection.^50^ It has been observed that the mature biofilm is found to be less sensitive to antifungal drugs, and microbes released from the biofilm are found to be highly virulent as compared to free floating cells in a murine model of disseminated candidiasis.^51,52^ Hsp90 protein regulates both dispersions of biofilm and antifungal drug resistance mediated by biofilm.^42^ Since we could find a role of Hsp90 in azole drug resistance in *pol32*ΔΔ, further we determined its ability to form biofilm on polystyrene surfaces. Biofilm formation was visualized by crystal violet staining and confocal scanning laser microscopy (CSLM) (Figure 8). More presence of crystal violet-stained particles on polystyrene surface and followed by the optical density measurement at 570 nm suggested comparatively better biofilm formation ability of *pol32*ΔΔ than the WT and *pol32*ΔΔ::*POL32 C. albicans* cells (Figure 8a). We further characterized the morphology of the biofilm by confocal scanning laser microscopy (CSLM), using silicone squares as the base. As depicted in Figure 8b, the wild-type strains formed a uniform thin layer of biofilm, whereas the *pol32* null strain developed compact but aggregated biofilm.

### Pol32 protein is essential for *C.* *albicans* virulence and systemic candidiasis development

**Figure 9.**
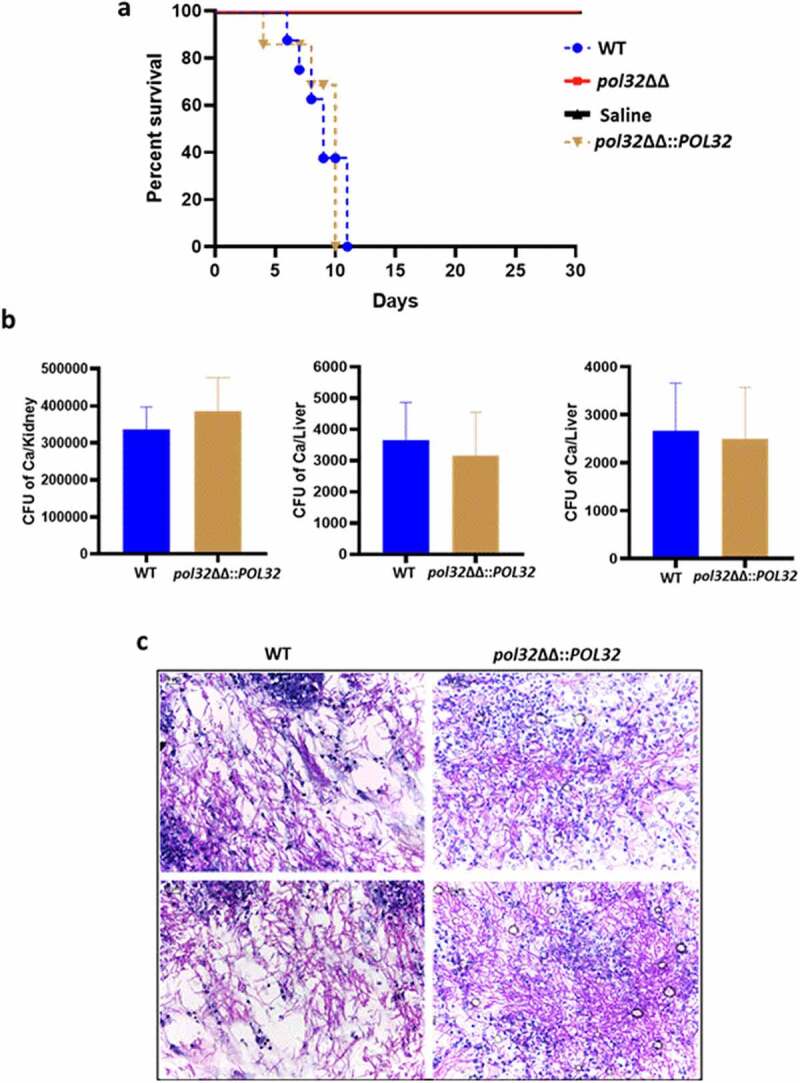
Mice model of systemic candidiasis. a. Male BALB/c mice of 6–7 weeks of age were inoculated either with WT or *pol32*ΔΔ or *pol32*ΔΔ::*POL32* (5x10^6^ CFU) *C. albicans* strains intravenously along with saline control and monitored their survival for 30 days. The survival curve was plotted using Graph pad prism 8.0 software. b. The murine kidney, liver, and spleen were collected and fungal burden was measured in all these organs by CFU determination. c. The kidneys of WT and *pol32*ΔΔ::*POL32* mice (n = 2) were stained with PAS staining and images were captured in a light microscope with 40X magnification.

Although Pol32 is a non-essential subunit of Polδ in *C. albicans*, its deficiency caused genomic instability, altered cell shape and size, reduced filamentation, and lowered virulence gene expression. All of these observations suggest that Pol32 could be an important protein required for *C. albicans* pathogenesis. Additionally, the intra-species diversity among the clinical strains of *C. albicans* has been found to result in differential virulence.^18^ To determine Pol32’s role in disease causing ability, we used a mouse model of systemic disseminated infections by the intravenous challenge of *C. albicans* cells. In this model, animals die due to severe sepsis with the highest fungal load in the kidneys; the phenotype mimics the severe cases of human fungal infections. The BALB/c male mice (n = 6) were injected with a fungal dose of 5 × 10^6^ CFU of *pol32*ΔΔ *C. albicans* cells per mouse via the lateral tail vein and monitored survival for 30 days (Figure 9). Similarly, three sets of mice (n = 6 x2) were challenged with the same CFU of WT and *pol32*ΔΔ::*POL32 C. albicans* cells and the same volume of saline as controls. Mice who suffered due to severe candidiasis succumbed or were sacrificed based on the severity of humane endpoints. While the mice challenged with WT and *pol32*ΔΔ::*POL32 C. albicans* succumbed to infection within 11 days (5–11 days) of inoculation, all mice challenged with the *pol32*ΔΔ strain survived and did not show any noticeable symptoms (Figure 9a). The experiment of mice challenged with *pol32ΔΔ C. albicans* strain was repeated and the same result was obtained (Supplementary Figure 7). The CFU analysis of the vital organs such as the kidney, liver, and spleen excised out of the WT challenged group of mice confirmed the presence of *C. albicans* cells (Figure 9b). Fungal load in the kidney tissue of suffered mice was very high (~3-5 × 10^5^ cells) in comparison to that in the liver and spleen (~500 cells). Histopathology of PAS-stained kidney sections also showed a heavy load of *C. albicans* cells in the kidneys of mice infected with WT and *pol32*ΔΔ::*POL32 C. albicans* (Figure 9c). Our virulence-challenged analyses demonstrated that the *pol32*ΔΔ *C. albicans* strain is avirulent, thus the Pol32 subunit of Polδ contributes significantly to various aspects of the life cycle of *C. albicans* and its pathogenesis. This attenuated strain can be explored further to develop a whole-cell vaccine against *C. albicans* or any other fungal or microbial species infections.

## Discussion

**Figure 10.**
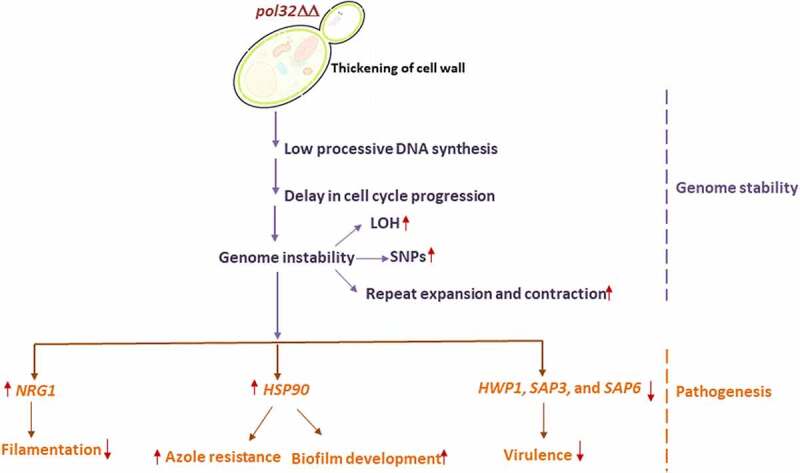
Lack of Pol32 links genome instability with *C. albicans* pathogenesis. It is a pictorial representation of the consequences of the deletion of *POL3*2 gene in *C. albicans*. Loss of Pol32 subunit renders replication stress by decreasing the processivity of Polδ, thereby inducing genome instability and delay in cell cycle progression. Critical hyphal and virulence genes get dysregulated to result in reduced filamentation, azoles resistance, enhanced biofilm formation, increased cell wall thickness with altered composition, and avirulency.

Being Polδ an essential replicative DNA polymerase, its fidelity and processivity are critical for the proper functioning of a eukaryotic genome as well as that of a cell. Abnormal activity of Polδ increases mutagenesis in yeast and tumorigenesis in mice and humans.^5,53^ Despite Pol32 playing important roles in fidelity, stability, and processivity of the Polδ holoenzyme and it is indispensable for cell survival in certain organisms; incongruously, in comparison to other subunits, its role has not been appreciated, but rather considered to be a mere accessory subunit. Therefore, in this study by using a pathogenic yeast *C. albicans* as a model system, we further explored the critical roles of Pol32 in the life cycle of *C. albicans* and for the first time, we demonstrated that the activity of Pol32 links genome stability with virulence (Figure 10).

According to the *Candida* genome database, Polδ possesses genes encoding all three subunits: *POL3* (C7_02790C_A/B and orf19.5182), *POL31* (C5_04740C_A/B and orf19.3960), and *POL32* (C1_05850W_A/B and orf19.2465). Similar to *S. cerevisiae*, human p12 homologue is absent in *C. albicans* as well. Thus, CaPolδ most likely is a holoenzyme of three subunits. Assuming that the Pol32 homologue of *C. albicans* will also be dispensable for cell survival, first, we generated a homozygous knockout by deleting both the alleles of *POL32* gene in SC5314 strain and the derived *pol32ΔΔ* cells survived and grew similar to its parental isogenic strain (**Supplementary Fig. 1C**). But we were unsuccessful in generating heterozygous deletion of *POL3* and *POL31* genes. Thus, both the alleles of *POL3* and *POL31* could be essential for cell survival in *C. albicans*. The *POL32* deficiency in *C. albicans* and *S. cerevisiae* caused growth retardation at high temperatures and in the presence of genotoxic agents (HU, UV, Cisplatin, MMS, and TBHP). Thus, consistent with earlier findings, Pol32 in yeasts plays a critical role in DNA replication and repair. To our surprise, unlike in *S. cerevisiae* where the N-terminal domain alone plays a vital role in the functioning of ScPol32,^7^ this is the first direct evidence to show that the essentiality of CaPol32 function is also PIP motif dependent. Accordingly, more accumulation of fragmented DNA and delayed cell cycle progression in *POL32* deficient and *pol32*ΔΔ::*POL32* F398A, F399A strains of *C. albicans* upon HU treatment were observed. DNA Polδ is inefficient in carrying out DNA synthesis against UV damages; however, Polζ with Pol31-Pol32 (Polζ4) complex carries out an efficient bypass of UV lesions.^10^ Since Polζ does not possess any PIP motif,^9,12^ the PIP motif of CaPol32 could be involved in the recruitment of Polζ4 to the stalled replication fork and essential for UV damage bypass or repair in *C. albicans*. Although the loss of *pol32* reduces UV-induced mutagenesis by Polζ in *S. cerevisiae*,^54^ the PIP motif of Pol32 during lesion bypass in *S. cerevisiae* is not critical, again it suggested a diverged mechanism of lesion bypass involving Pol32 in *C. albicans*. Intriguingly, contrary to *S. cerevisiae pol32*Δ,^11^ we found an increased rate of LOH without any addition of genotoxic stressors in both *C. albicans pol32*ΔΔ and a strain carrying PIP mutant of *POL32*. Thus, Pol32 is mostly an antimutator in *C. albicans*. To get more insights into the antimutator phenotype of Pol32, whole-genome sequencing of WT and *pol32*ΔΔ strains of *C. albicans* was carried out and compared with the publicly available reference sequence of SC5314 strain. Over time, our laboratory WT strain has accumulated base substitutions and indels, however, these variations have not altered the native phenotypes such as morphological switching, sensitivity to fungal drugs, virulence, etc. Such microscale changes are very common and have already been demonstrated by whole-genome sequencing of *C. albicans* isolates passaged both *in vitro* and *in vivo* during laboratory culture and in the mammalian host.^25^ YMAP clearly showed base substitutions but no aneuploidy in the WT strain. In addition to these genetic changes, the *pol32*ΔΔ strain accumulated 2125 indels and 6991 SNPs, and these could be specific to the Pol32 affecting Polδ’s function. To ensure that these genetic changes are specific to the function of Pol32 and were not obtained during the generation of deletion strain, we sequenced the whole genome of similarly generated mutant strains of *C. albicans* defective in DNA polymerase- zeta and epsilon along with the *pol32ΔΔ* strain. Our comparative analysis revealed that the rate and position of indels and SNPs are altogether different in different strains that suggest the specific role of respective DNA polymerases in genome instability (data not shown). Since we observed 2 folds more indels in homo- or hetero-polymeric repeat regions of the chromosome and as such regions are intrinsic replication blockers, it suggests that reduced processivity of Polδ in *pol32ΔΔ* could be the rationale behind the higher rate of such deletions and insertions. Genetic evidence suggests a division of labor at the replication fork where Polδ is mostly involved in lagging strand and Polε in leading strand DNA synthesis. Okazaki fragment maturation often leads to the generation of genetic variations.^55^ Higher accumulation of heterozygous indels (2096 out of 2125) could be due to the role of Polδ in lagging strand DNA synthesis. We only observed a segmental aneuploidy in Chr 7 of *pol32ΔΔ* which may cause gene family contractions. Segmental aneuploidy is often associated with developmental abnormalities, reduced fitness, and occasionally a significant growth advantage over euploid cells under some conditions, most likely due to different gene dosages causing imbalances in protein stoichiometry.^56,57^ Among the chromosomes, Chr 7 and Chr R carry two MRS sequences flanking the centromere, thus they are vulnerable to being either contracted or expanded during a compromised replication condition. In budding yeast, deletion of the *POL32* gene caused a 3.3-fold increase in the contraction rate of GAA repeats independently of fidelity of Polδ.^58^

Since the loss of *pol32* caused replication stress and genome instability that can impact the life cycle of *C. albicans*, various pathogenic attributes of the *POL32* deficient cells were determined. Morphogenesis of *C. albicans* is one of the virulence determinants. In the presence of replication inhibitor and DNA damaging agents, we did not find any significant difference in the morphological transition of both Pol32 proficient and deficient strains of *C. albicans*; however, in the presence of serum containing media and spider media, reduced filamentation in *pol32*ΔΔ with smaller germ tube was found. This difference in morphology is also associated with somewhat reduced expression of virulence genes such as *ECE1, SAP3, SAP6*, and *HWP1*, and overexpression of transcriptional repressor *NRG1*. In fact, the role of *NRG1* in the downregulation of *ECE1* and *HWP1* has been already deciphered.^59^ Since *NRG1* is belonged to Chr 7, whether the segmental aneuploidy in Chr 7 of *pol32*ΔΔ led to the up-regulation of *NRG1* requires further investigation. However, the genome browser did not show any mutations in *NRG1* of the *pol32*ΔΔ strain. The *pol32* defective strain showed azole drug resistance but amphotericin B hypersensitivity. The mechanism of azoles drug resistance of *pol32*ΔΔ cells appears to be multifactorial. An altered cell shape, cell size, cell wall composition with more chitin and glucan content, lowered expression of *ERG3*, and hyper-expression of *HSP90* and *HSP30* could determine azoles drug resistance of *pol32*ΔΔ strain. A reduced level of *ERG3* causing azole drug resistance in *C. albicans* has already been demonstrated.^44,60^ *HSP90* functions via Tor1 signaling, and rapamycin inhibits the hyper-activation of Tor1. Since *pol32*ΔΔ is sensitive to rapamycin, we concluded that the Tor1 pathway is not involved in azoles drug resistance. However, hypoacetylated Hsp90 is involved in azoles drug resistance as geldanamycin and trichostatin A sensitized *pol32*ΔΔ strain to azole. Thus, we concluded that *HSP90* could involve in azoles resistance in *C. albicans* independent of the Tor1 pathway. The *pol32*ΔΔ strain also formed a better biofilm than the wild type *C. albicans* and a link between *HSP90*, azole drugs resistance, and biofilm has been shown.^42^ Replication and oxidative stresses are known to up-regulate *HSP90* expression, although antifungal drugs enhance oxidative stress, this overexpression of *HSP90* is most likely due to the replication stress in *pol32*ΔΔ strain.^61,62^ The stress response by Hsp90 is regulated by several factors like affinity of ATP binding and hydrolysis, interactions with co-chaperones, and post-translational modifications. A recent study suggested the role of lysine deacetylases (KDACs) like Hda1 and Rpd3 in regulating Hsp90 function in azole resistance in *C. albicans* and *S. cerevisiae*. Inhibition of lysine deacetylases by molecules like trichostatin A leads to the accumulation of hyperacetylated Hsp90. Hyperacetylation will prevent the binding of co-chaperons like calcineurin and other client proteins thereby inhibiting Hsp90’s function.^63^ Since this study finds increasing efficacy of azole drugs by geldanamycin and trichostatin A, it again advocates the use of a combinatorial therapy by targeting the molecular chaperone Hsp90 and its upstream and downstream targets to enhance azoles drug efficacy. Further, we used an animal model to check the pathogenic potential of the *pol32*ΔΔ strain to develop systemic candidiasis, and it was completely attenuated and the infected mice survived. This attenuation of the *pol32*ΔΔ strain could be because of altered cell structure, reduced filamentation, and a lowering in the expression of certain virulence genes. We found hyperexpression of *NRG1* in the *pol32ΔΔ* strain and previous studies have already described the efficacy of a genetically engineered *C. albicans tet-NRG1* strain as an experimental live attenuated vaccine against hematogenously disseminated candidiasis.^64^ *ECE1* and *SAP3* genes were shown to be involved in host cell adhesion and penetration; and decreased virulence of the respective null strains has already been demonstrated.^38,65^ We presume *POL32* could be an important virulence gene and thus, the *pol32*ΔΔ strain could be a potential vaccine candidate. In summary, we conclude that although Pol32 is dispensable for cell survival, it plays a critical role in stabilizing the genome, morphological transition, and candidiasis development. Since the PIP motif of Pol32 is essential for its function in *C. albicans*, we propose to develop a small-molecule inhibitor to block its interaction with PCNA as an antifungal drug. In addition, as Pol32 is essential for *C. albicans* pathogenesis and no approved vaccine against any fungal diseases is available yet, we suggest exploring the *pol32* null strain as a whole-cell vaccine against candidiasis and other fungal infections.

## Materials and methods

### Ethics statement

Mice experiments were conducted in strict accordance with the guidelines of the Institutional Animal Ethical Committee. Experimental animal protocols were fully approved by the committee and given the ethical Permit Number ILS/IAEC/133-26AH/AUG-18. Necessary precautions were taken to minimize animal suffering and to ensure the highest ethical and humane standards.

### Animal, reagents, and chemicals

In-house breed strains of BALB/c male mice of 5–7 weeks old were maintained in individually ventilated cages under the standard condition with ad libitum. The oligonucleotides used in this study were procured from Integrated DNA Technologies (IDT, USA). *C. albicans* (SC5314) and *S. cerevisiae* (EMY74.7) were used as wild-type yeast strains and various knockouts were derived from these parental strains (Table 3). YPO69 is a *pol32* null strain of *S. cerevisiae* that was derived from EMY74.7. Fetal bovine serum-South American origin (# P30-3302) from PAN Biotech, GmbH, Germany, YPD media (# G037) from HIMEDIA, India, enzymes required for cloning from NEB, genotoxic agents like HU (#102023), Cisplatin (#198872), TBHP (#190049), and MMS (#205518) from MP Biomedicals, and antifungal drugs such as fluconazole (Cat #F8929), ketoconazole (Cat# K1003), miconazole (#PHR1618), geldanamycin (#G3381), trichostatin A (#T8552), and Aniline blue (#B8563) from Sigma were procured. Periodic Acid Schiff (PAS) Stain Kit (Mucin Stain) (#ab150680) was obtained from Abcam, Cambridge, USA. The yeast strains were grown in YPD media without or with maltose or various synthetic drop-out media as required at 30°C.

**Table 3.** Various strains used for this study.

Strain	Description /Genotype	Source/Reference
SC5314	*C. albicans* Wild type (WT)	(Braun, *et al. ^23^*)
CNA25	Homozygous knockout of *POL32* gene in SC5314 (*pol32ΔΔ*)	in this study
CNA39	Re-integration of *POL32 in* CNA25 (*pol32ΔΔ::POL32*)	in this study
CNA45	Re-integration of *POL32* gene with PIP mutation in CNA25 (*pol32ΔΔ::POL32* F398A, F399A)	in this study
CNA126	Heterozygous knockout of *URA3* gene in SC5314 (*ura3Δ*/URA3)	In this study
CNA127	Heterozygous knockout of *URA3* gene in CNA25 (*pol32ΔΔ, ura3Δ*/*URA3*)	In this study
CNA139	Heterozygous knockout of *URA3* in CNA25 (*pol32ΔΔ:POL32 ura3Δ*/URA3)	In this study
CNA140	Heterozygous knockout of *URA3* in CNA25 (*pol32ΔΔ::POL32 PIP ura3Δ*/URA3)	In this study
EMY74.7	*S. cerevisiae* Wild type	^3^
YPO69	Deletion of *POL32* gene in EMY74.7	^3^

### Bioinformatics analysis

The putative Pol32 ORF of *C. albicans* was aligned with ScPol32, SpCdc27, and Hsp68 by using Clustal Omega (https://www.ebi.ac.uk./Tools/msa/clustalo/). The online modeling database SWISS-MODEL (https://swisssmodel.expasy.org) was used to generate the model structure of CaPol32. The model structures of various PIP domains were predicted using the PEP-FOLD 3.0 server (https://bioserv.rpbs.univ-paris-diderot.fr/services/PEP-FOLD3. The model structures generated were aligned with the PIP peptide sequence from p68 (1U76) without or with PCNA.

### Generation of Pol32 constructs

The *CaPOL32* gene and its ORF were PCR amplified in a 50 μl reaction volume containing 50 ng of genomic DNA, 1.5 mM MgSO_4_, 1x buffer, 250 μM dNTPs, 2 U of Q5 DNA polymerase, and 10 pmol of each of the primer pairs NAP37 (5’-CCG GAA GCT TAG CTG CCC CTG TTG CTG CTC-3’) – NAP38 (5’-GGC CGA ATT CTA ACC ATA ATT TCA TTA GTA ATG TC-3’) and NAP39 (5’-GGC CAA GCT TGG ATC CAC ATA TGA CGA TGA GTA GTT CTG CTG ATG-3’) – NAP40 (5’-CCG GGA ATT CGG ATC CTC ACT TTT TCT TTC CAA AAA AAC-3’), respectively. The amplified PCR products were purified, digested with HindIII and EcoRI enzymes, and cloned into pUC19. The BamHI fragment containing Pol32 ORF was further subcloned into the BglII site of a yeast expression vector for protein purification. Similarly, the *POL32* gene with PIP mutation (F398A, F399A) was amplified from SC5314 genomic DNA by using primers NAP404 (5’-CCG GGG TAC CCT GTT GCT GCT CAA CCT G-3’) and NAP463 (5-GGCC CTC GAG CAC TTT TTC TTT CCT GCA GCA CTC ATT AAT G-3’). The amplified product was purified, digested with KpnI and XhoI restriction enzymes, and cloned into the KpnI-XhoI site of a modified pSFS2 vector. The Pol32 ORF with PIP mutation (F398A, F399A) was further amplified from the resultant clone using primers NAP39 (5’-GGC CAA GCT TGG ATC CAC ATA TGA CGA TGA GTA GTT CTG CTG ATG-3’) and NAP336 (5’-GAA ATC CAG ACA GTC GAG-3’). The amplified product was digested with BamHI and cloned into the BglII site of a yeast expression vector. For *S. cerevisiae*, ScPol32 ORF and its PIP motif mutant (F344A, F345A) were amplified from EMY74.7 genomic DNA using forward primer NAP590 (5’-CCG GGG ATC CAC ATA TGG ATC AAA AGG CGT C-3’) and reverse primers NAP591 (5-CCG GGG TCC TTA TTT TGC CTT TCT TTT G-3’) and NAP813 (5’-CCG GGG ATC CTA TTT TGC CTT TCT TTT TGG CAG CGC TTT CC-3’), respectively. The amplified PCR products were cloned into the BamHI site of a 2µ-Sc-*ADH1*p plasmid vector. All the plasmid constructs were authenticated by DNA sequencing.

### Protein expression and purification

Our yeast expression vector provides an option to express protein under the GAL-PGK promoter with an amino-terminal GST-fusion in a protease deficient *S. cerevisiae* strain.^66^ While *C. albicans* Pol32 proteins were expressed in yeast, PCNA was expressed in *E. coli* BL21 DE3 cells as GST fusions and purified using glutathione-Sepharose 4B beads as described previously.^67,68^ To obtain untagged proteins, GST-fused proteins bound to glutathione-Sepharose beads were treated overnight at 4°C with PreScision protease to cleave the GST tag from the Pol32 or PCNA protein. All the purified proteins were stored at −80°C. Some of the uncleaved protein beads were saved for pull-down assay when required.

### Generation of *C.* *albicans* knockout strains

The *SAT1* flipper strategy was used to create a null mutant of the *POL32* gene in the SC5314 strain of *C. albicans* as described before.^23,69^ Both copies of *POL32* genes were deleted sequentially by transforming a linear DNA consisting of a *SAT1* marker gene flanked by upstream and downstream sequences of the *POL32* gene (Supplementary Figure 1A and B). Two deletion constructs were generated by PCR cloning each with the same downstream flanking region but differed in having upstream sequences. The downstream fragment was amplified with primers NAP311 (5’-GGC CGA GCT CCT CGC TCT GGA GAA CAA GAA G − 3’) and NAP407 (5’-CCG GGA GCT CGG TGA CCA AAT TTC ATT GC-3’) from the *POL32* gene template construct and cloned into SacI site of pSFS2. The upstream flanking regions −342 to +109 bp and −342 to +249 bp of *POL32* were PCR amplified with a common forward NAP404 (5’-CCG GGG TAC CCT GTT GCT GCT CAA CCT G-3’) and two reverse primers NAP405 5’-GGC CCT CGA GCA TGG ATG TTG AGT TG-3’) and NAP406 (5’-CCG GCT CGA GCA AAT CTG ACT CCA AAT C-3’), were digested with KpnI – XhoI and cloned into the construct containing downstream sequence to generate pNA1554 and pNA1559 deletion constructs, respectively. In the first round, the cassette was amplified by PCR from the deletion construct pNA1554 using forward primer NAP404 and reverse primer NAP407, and the amplified DNA was transformed into SC5314, and transformants were selected on a YPD-agar plate containing NAT (100 µg/ml nourseothricin). Integration of cassette at the correct locus was confirmed by PCR with primers NAP404 and NAP336. For recycling of the *SAT1*, PCR positive colonies were grown on YPM media for 48 hr, and the loss of the cassette was confirmed by their inability to grow on NAT containing plate. For generating homozygous deletion strain (*pol32ΔΔ*), the deletion cassette from pNA1559 was transformed to *POL32/pol32Δ* strain and a similar screening approach was used. The colonies were then cured for three days in YPM broth and streaked on YPM and YPM+NAT plates to confirm the absence of NAT cassette retention. Finally, the deletion of the *POL32* gene was confirmed by PCR using the primers NAP39 and NAP40. Amino acids from 31 to 339 of CaPol32 orf (out of 403 amino acids) were disrupted in heterozygous deletant, whereas 31–339 and 81–339 amino acids were deleted in both the chromosomal copies in homozygous *pol32* deletant. Therefore, we detected two PCR fragments of 308 and 448 base pairs corresponding to the deletion of both alleles in homozygous deletion in comparison to single-copy deletions. To integrate wild-type and PIP mutant of Ca*POL32* into the *pol32ΔΔ* strain of *C. albicans*, full-length *POL32* genes without or with F398A, F399A mutation was amplified by NAP404 forward and NAP462 (5’-GGCC CTC GAG ACA AAA CTA AAT TTC ACT TTT TCT TTC C-3’) or NAP463 (5’-GGCC CTC GAG C ACT TTT TCT TTC CTG CAG CAC TCA TTA ATG-3’) reverse primers. The PCR amplified products were digested with KpnI – XhoI and cloned into pNA1554 to generate integration constructs pNA1612 and pNA1620, respectively. The cassettes were amplified from the integration constructs pNA1612 and pNA1620 using forward primer NAP404 and reverse primer NAP407, and the linearized fragments were transformed into the *pol32ΔΔ* strain of *C. albicans*, the transformants were selected in YPD+NAT plates, and the same methodology was followed till NAT curing. The integration was confirmed by PCR using NAP39 and NAP40.

### GST-pull down assay

GST-CaPol32 and CaPol32 (F398A, F399A) protein bound to glutathione sepharose beads were mixed with 0.5 µg CaPCNA and incubated overnight. The beads were then washed thrice with 0.3 ml of equilibration buffer (50 mM Tris-HCl pH 7.5, 150 mM NaCl, 5 mM dithiothreitol, 0.01% NP-40, 10% glycerol) to remove unbound proteins. The bound proteins were finally eluted in 40 µl of SDS loading buffer. Various fractions were resolved on 12.5% SDS-PAGE, followed by western blotting, and detection by using an in-house prepared anti-CaPCNA antibody (1:7000 dilution) and an HRP conjugated secondary antibody (Cat#69265, Novagen) (1:10000 dilution).

### ITC assay

The purified CaPol32, CaPol32 PIP (F398A, F399A), and CaPCNA proteins were dialyzed overnight in a buffer containing 20 mM HEPES (pH7.4) and 150 mM NaCl. ITC experiments were performed using a MicroCal PEAQ-ITC system (Malvern Panalytical) at 25°C with a 20 μM concentration of CaPol32 or CaPol32 (F398A, F399A) loaded into the sample cell and 250 μM CaPCNA was injected into the cell by a given syringe. Twenty five consecutive injections of 1.5 μl each were made at an interval of 120 s. A control experiment was performed by monitoring any heat dilution of ScPol32 by buffer alone injection. Similarly, heat exchange by a buffer-buffer titration was also estimated and the value was subtracted from the respective individual experiment. Binding isotherms were fitted into a one-site binding model using the MicroCal PEAQ-ITC analysis software.

### Growth curve

Various strains of *C. albicans* and *S. cerevisiae* were grown overnight in YPD broth. The overnight cultures of various strains were diluted to an OD_600_ = 0.01 with fresh 10 ml YPD media and allowed to grow at 30°C. Absorbance was measured at OD_600_ nm at every 2 hours interval for 14–15 hr. For antifungal drug susceptibility, a similar growth curve analysis was carried out in YPD medium containing 6 μM fluconazole. The OD values were plotted using a Graph pad prism v8.0. The experiments were performed twice with biological duplicates.

### Sensitivity assays

Overnight grown cultures of various strains of *C. albicans* and *S. cerevisiae* were diluted in YPD liquid media to achieve an equal number of cells. They were further serially diluted by 10 folds and spotted on YPD plates containing the indicated concentration of HU, MMS, cisplatin, and TBHP. For UV sensitivity, the plates were exposed to different doses of UV and wrapped with aluminum foil. All the plates were incubated at 30°C for 2 days and images were captured. For the temperature sensitivity test, spotted plates were incubated at 16°C, 30°C, 37°C, and 42°C for 2 days and then photographed. For quantitative survival experiments, logarithmically growing cells were diluted to ~500 cells/ml, from which 200 μl was spread onto YPD plates with or without DNA-damaging agents at indicated concentrations. For UV treatment, the plates were exposed to UV irradiation at different doses. After 2- to 3-days of incubation, the number of colonies on each plate was counted and percent survival with respect to untreated was determined.

### Pulsed-field gel electrophoresis

DNA plugs were prepared using Bio-Rad CHEF Yeast Genomic DNA Plug Kit as per the manufacturer’s instruction. The chromosomal separation was performed in 1.2% agarose gel with 0.5 X TBE buffer using a CHEF Mapper XA system. The running condition was voltage-6v×cm-1, switch time-60 to 120 s, angle-120° for 36 hr followed by voltage-4.5 v× cm-1, switch time-120s to 300s, angle- 120° for another 12 h. The gel was then stained with ethidium bromide and the image was captured in a ChemiDoc system.

### Alkali agarose gel electrophoresis

Overnight grown cultures of various strains of *C. albicans* were diluted in 100 ml fresh YPD media to an OD_600_ = 1 and allowed to grow at 30°C for 2 hr. HU (100 mM) was then added and allowed to further grow for another 45 mins at 30°C. Cells were harvested and the pellet was washed twice with sterile distilled water, resuspended in 100 ml of fresh YPD media, and allowed to grow. Subsequently, cells were harvested at 0, 6, 12, and 24 hr of recovery periods, and the total genomic DNA was extracted. The genomic DNA was resolved by alkaline agarose gel electrophoresis as described previously.^68^ For visualization, the gel was stained with ethidium bromide and the image was captured.

### Loss of heterozygosity assay

To calculate chromosomal instability, a copy of the *URA3* gene was deleted from the SC5314 and its derivative strains to generate heterozygous *ura3Δ/URA3* strains. A similar method as described for *POL32* gene knockout was used to delete a single copy of the *URA3* gene. The upstream flanking region was amplified with primers NAP746 (5’-CCG GGG TAC CGT CAT TCC TCT TG-3’) and NAP747 (5’-GGC CCT CGA GAC TGG TGA GGC ATG AG −3’) from the genomic DNA and cloned into KpnI and XhoI sites of pSFS2. The downstream fragment was amplified by PCR with a forward NAP748 (5’-GGC CCC GCG GGA TGC TGG TTG GAA TG −3’) and reverse primers NAP749 (5’-CCG GGA GCT CGA AGA TTA TAA TGA TGT TC −3’), was digested with SacII-SacI, and cloned into the construct containing upstream sequence to generate pNA1760 *URA3* gene deletion construct. The deletion cassette was excised out by KpnI–SacI digestion, and transformed into various *C. albicans* strains. The heterozygous deletion was confirmed by PCR using NAP746 and NAP749 primers. The strains were grown in YPD medium at 30°C overnight. The cells were then harvested, washed twice with sterile distilled water, and diluted to get an appropriate number of cells. Around 50–200 cells were spread on SD agar plates without or containing 100 mg/ml FOA. All the plates were incubated at 30°C for 3–4 days. The colonies of each strain were counted for SD and SD + FOA agar plates. The complete loss of *URA3* in these strains in the presence of 5FOA was verified by growing them on media lacking uracil. The experiments were performed in biological triplicate with technical duplicates.

### Cell cycle analysis

*C. albicans* cells were synchronized by inoculating an isolated colony in 5 ml of YPD broth from freshly streaked cells and incubated at 30°C for 16 hr in shaking condition. Synchronized cells were diluted in fresh YPD media without or with 4 mM HU and were grown at 30°C for 150 min. The cells were collected at various time points and washed twice with 700 μl of sterile distilled water. The cell pellet was resuspended in 300 μl of sterile distilled water and fixed with 700 μl of 100% ethanol at −20°C for 12 hr. Cells were harvested, washed twice with 1 ml of 50 mM of Sodium Citrate buffer, pH 7.4 for 10 mins at 25°C. The cell pellet was finally resuspended in 1 ml of 50 mM of Sodium Citrate containing 0.25 mg/ml RNase A and incubated at 37°C for 12 hr. Further, 25 μl of 20 mg/ml protease K was added and incubated for another 1 hr. The cell pellet was harvested and resuspended again in 500 μl of 50 mM sodium citrate buffer. The staining was done with 1 μM of SYTOX green by overnight incubation at 4°C in dark. The cells were transferred to FACS tubes, washed with 500 μl Sodium Citrate buffer, and analyzed by Flow cytometry. *C. albicans* cells were distinguished from debris by plotting SSC-A versus FSC-A, followed by singlet discrimination using FSC-H versus FSC-A, and finally singlet cells were visualized by blue laser excitation (488 nm).

### Whole-genome sequencing

Genomic DNA libraries were constructed in alignment with microbial whole-genome sequencing recommendations of the Nextera^TM^ DNA flex library preparation Kit from Illumina Inc. Briefly, 250 ng of DNA was used for tagmentation, followed by the addition of dual indices and adapters to generate the respective libraries. The cleaned libraries were quantitated on a Qubit^R^ fluorometer and appropriate dilutions were loaded on a D1000 screen tape to determine the size range of the fragments and the average library size. The average library size was 412 bp and 400 bp for WT and *pol32ΔΔ* strains of *C. albicans*, respectively. After QC clearance of the libraries, they were diluted to 4 nM, pooled, spiked with 5% PhiX pre-made library from Illumina, and loaded on a MiSeq v3 kit. Sequencing was performed for 2 × 150 cycles. The original raw data obtained from Illumina MiSeq as FASTQ files (containing paired-end reads sequences) was processed to obtain adapter free reads from MiSeq. The quality of the reads was checked using FastQC and the reads were trimmed using Trimmomatic. De-novo assembly of filtered data was performed using Shovill, which has SPAdes assembler at its core along with additional pre- and post-processing steps to improve the assembly. The raw reads were mapped to the publicly available reference genome of *C. albicans* SC5314 (assembly ASM18296 v3) using bowtie2 to calculate overall genome coverage. The overall alignment rate was found to be satisfactory to proceed with variant calling (94.91% for wild type and 95.68% for *pol32ΔΔ*). Variant calling was used to extract and filters SNPs and Indels. This was followed by filtering the variants to get the variants with high confidence. For filtering the variant, standard hard-filtering recommendations of GATK were used (https://gatk.broadinstitute.org/hc/en-us/articles/360035890471-Hard-filtering-germlineshortvariants). The filters used for SNPs are QD<2.0; FS>60; MQ<40; SOR>10 and for Indels, the filters used are QD<2.0; FS>200; SOR>10. Along with this, an additional filter of the depth-read (DP<50) was also applied. SnpEff database was used for variant annotation. The VCFPolyXutility of Jvarkit was used on the annotated variants to find out the number of repeated reference bases around the variant position

### YMAP analysis

Copy number and allele status of various *C. albicans* strains were visualized using YMAP (https://lovelace.cs.umn.edu/Ymap/). The raw FASTQ files of both wild-type and *pol32*ΔΔ strains were uploaded into the YMAP server to map bonafide CNVs after applying filters to remove GC-content bias and chromosome-end bias. The reference strain used for this study is SC5314_A21-s02-m08-r09.

### Morphology of *C.* *albicans*

Overnight grown *C. albicans* cells were diluted to an OD_600_ = 0.05 with 5 ml YPD medium with or without 5 mM of HU, 0.002% MMS, 0.5 mM cisplatin, and 0.004% TBHP and grown at 30°C. After 4 h of growth, cells were observed under the Leica microscope with 40X magnification. For colony morphology, 100 μl of the overnight grown culture of WT and *pol32*ΔΔ *C. albicans* containing an equal number of cells were spread on spider agar media (1% Nutrient Broth, 1% Mannitol, and 0.2% of K_2_HPO4) and YPD-FBS agar media (1% yeast extract, 1% peptone, 4% dextrose, and 10% FBS). Plates were incubated at 37°C, and images were taken after mentioned duration of incubation. Similarly, for germ tube development, *C. albicans* cells were cultured in a YPD medium containing 10% FBS at 37°C for 1 hr and observed under the microscope. The lengths of germ tubes (n = 24) were measured for each strain using Image J software and represented in a graph using Graph pad prism 8.0 software.

### Antifungal drug susceptibility assay

The pre-cultures of the various strains of *C. albicans* were diluted to an OD_600_ of 1. The samples were further serially diluted by 10 to 10000 folds and spotted on YPD plates without or containing different concentrations of fluconazole, ketoconazole, miconazole, 5-flurocytosine, 5-fluorouracil, amphotericin B, SDS, CFW, CaCl_2_, berberine, rapamycin, and Congo red. For Hsp90 inhibition, cells were spotted on YPD plates containing geldanamycin or trichostatin A and without or with 6 μM fluconazole. All the plates were incubated at 30°C for two days and images were captured. Similarly, to check the sensitivity in the liquid medium, the strains were cultured in fresh YPD media with or without fluconazole (6 μM), geldanamycin (6 μM), trichostatin A (6 μM), and a combination of fluconazole and geldanamycin or trichostatin A. The OD values were plotted using Graph pad prism v8.0. The experiment was performed twice with biological duplicates.

### Determination of chitin content by flow cytometer

The chitin content in the cell wall of *C. albicans* was measured using a methodology described previously with a minor modification.^70^ Briefly, cells were harvested at their late logarithmic phase growth at 30°C, washed twice with sterile water, and re-suspended in sterile water to get about 10^6^ number of cells per ml. About 2.5 µg CFW was added to 1 ml of cells and incubated at 25°C for 15 min. Next, the cells were washed twice with PBS, resuspended in PBS, transferred to FACS tubes, and acquired in BD LSRFortessa™ Cell Analyzer – Flow Cytometers using UV laser (350 nm) with bandpass filter (450/50 nm). The mean fluorescence intensity obtained from stained and unstained yeast cells were analyzed and processed in the flowjo software.

### Berberine and Congo-red accumulation assay

The uptake and accumulation of berberine and Congo red compounds in various strains of *C. albicans* was measured by using FACS.^71^ The log phase cells were harvested, washed in PBS, and diluted to O.D._600_ of 0.1 in PBS containing 2% glucose. A final concentration of 25 µM berberine or Congo-red was added to the cell suspension, and incubated at 30°C for 1 hr in shaking condition. The cells were pelleted, washed with PBS, and resuspended in PBS. The cell suspension was taken up in FACS tubes and analyzed in BD LSRFortessa™ Cell Analyzer – Flow Cytometers using UV laser (350 nm) with bandpass filter (450/50 nm) or Blue laser (488 nm) with long pass mirror (610 nm). The mean fluorescence intensity obtained from stained and unstained yeast cells were analyzed and processed in the flowjo software

### Gene expression analysis by PCR

Overnight cultures of WT and *pol32ΔΔ* strains were harvested and total RNA was isolated using the Gene JET RNA purification kit as per the manufacturer’s instruction. About 2 µg of RNA was used to synthesize cDNA using high capacity cDNA reverse transcription kit from Invitrogen with the provided random primers. A total volume of 20 µl of qRT PCR reaction mixture was setup containing 50ng of cDNA, 10 pmol of forward and reverse primers, and 2x SYBR green qPCR Master mix. The qRT PCR cycling was carried out in Quant Studio 3 with fast cycle conditions including 95°C for 2 min followed by 40 cycles of 95°C for 5 sec denaturation and 60°C for 30 sec for annealing and extension. All the experiments were performed in biological duplicates with technical triplicates. The data obtained were analyzed using the 2^−ΔΔCT^ method. The gene expression of WT and *pol32*ΔΔ strain was normalized with *GAPDH*, the housekeeping gene and graphs were plotted using a Graph Pad Prism 8 software. Similarly, for semi-quantitative real-time PCR, 20 µL of reaction mixture was setup containing 50 ng of cDNA, 10 pmol of respective forward and reverse primers, 250 µM dNTPs, 1x Taq DNA polymerase buffer, and 1 U of Taq DNA polymerase. The PCR conditions used were initial denaturation at 95°C for 1 min, followed by 30 cycles of 95°C for 30 sec, 60°C for 30 sec and 72°C for 30 sec. The amplified products were resolved in 1.2% and images were captured in a gel doc system. The band intensity of the amplified products were analyzed using image J software. The band intensity of WT and *pol32*ΔΔ strain was normalized by the house keeping gene *GAPDH* and graphs were plotted using Graph Pad Prism 8 software. The primer sequences of various genes used for real-time PCR were given in Table 4.

**Table 4.** Primers used for real-time PCR.

Gene name	Forward primer	Reverse primer
*GAPDH*	5’-gaccgttgacggtccatcc-3	5’-catcggtggttgggactc-3’
*ALS1*	5’-cctatctgactaagactgcacc-3’	5’-acagttggatttggcagtgga-3’
*ALS3*	5’-cggttgcgactgcaaagac-3’	5’-gaccaacccaaaacagcattcc-3’
*HWP1*	5’-cagttccactcatgcaaccatc-3’	5’-gcaataccaataatagcagcaccg-3’
*ECE1*	5’-ccggcatctcttttaactgg-3’	5’-gagatggcgttccagatgtt-3’
*SAP3*	5’-gttactggtccccaaggtg-3’	5’-cttgtccttgaccagcttgac-3’
*SAP6*	5’-gtcaacgctggtgtcctc-3’	5’-gcaggaacggagatcttgag-3’
*NRG1*	5’-cacctcacttgcaacccc-3’	5’-gccctggagatggtctga-3’
*TUP1*	5’-ctcttggcgacaggtgcag-3’	5’-gtggtgacgccgtcttcga-3’
*CDR1*	5’-aaagatgacctcgtcagcaggttt-3’	5’-ccaattcccaatttcgaaggt-3’
*CDR2*	5’-tgttggtaccatttcatatttctgttg-3’	5’-aagagattgccaattgtcccata-3’
*MDR1*	5’-tcgttttagcaatggcgtttg-3’	5’-ccatgccctccaatgaacag-3’
*ERG3*	5’-tccagttgatgggttcttcc-3’	5’-ggacagtgtgacaagcgg-3’
*ERG11*	5’-ttacctcattattggagacgtgatg-3’	5’-cacgttctcttctcagtttaatttctttc-3’
*HSP90*	5’-aagtgctggtgctgacg-3’	5’-cttaccaccagcgttag-3’

### Transmission electron microscopy (TEM)

Ultrastructure of WT and *pol32*ΔΔ *C. albicans* cells were examined by TEM using a protocol described before.^72^ Briefly, similarly grown log-phase cultures were centrifuged at 5000 g for 5 min followed by washing with 1 ml of 0.1 M cacodylate buffer. The pellet so obtained was fixed with 1 ml of 2.5% glutaraldehyde for 30 min at 25°C. The fixed cells were washed again using cacodylate buffer followed by post-fixation using 200 μl of 1% osmium tetraoxide for 3 hr. The dehydration process was carried out with 50%, 70%, 90%, and absolute alcohol, each with 10 min of incubation. Next, the pellet was first resuspended in 500 μl of propylene oxide and then again resuspended in 1 ml of a mixture of propylene oxide and Spurr’s resin (1:1 ratio) for 3 hrs. The impregnation of samples was carried out in pure resin and incubated overnight at RT. Prior to sample embedment, the overnight pellet was centrifuged at 5000 g, the resin was discarded, and again resuspended in a resin mixed hardening reagent DMAE (Ted Pella, Inc.). The mixture was allowed to polymerize at 60°C for 48 hrs. The hardened sample was sectioned using Leica EM UC7 microtome. The sections were collected on the copper grid, stained with uranyl acetate for 30 min, followed by 3 times washings with distilled water, and allowed to dry for 2 h. Samples on the grid were visualized under JEM-2100Plus JEOL TEM imaging machine.

### Aniline blue assay

The β-glucan level of the fungal cell wall was measured by aniline blue assay.^73^ Briefly, 1 ml of overnight grown cells was harvested and washed two times with 1xTE buffer. The cells were resuspended in 1xTE buffer to obtain an OD_600_ of 0.2. About 500 μl cell suspension was treated with 100 μl of 6 M NaOH at 80°C for 30 min, followed by the addition of 2.1 ml of aniline blue mix (0.03% aniline blue, 0.18 M HCl, and 0.49 M glycine-NaOH, pH 9.5), and then vortexed. The tubes were again incubated at 50°C for 30 min followed by cooling at 25°C for 30 min. The fluorescence intensity (FI) was measured by taking the mixture in a 96 well black microplate using a plate reader at an excitation wavelength of 400 nm/slit and an emission wavelength of 460 nm/slit with a cutoff of 455 nm. The fluorescence intensity obtained from both the samples was plotted using Graph pad Prism v 8.0.

### Biofilm growth

*C. albicans* cells were grown in 24-well polystyrene plates containing YPD media with 10% serum for 24 hours at 37°C. Supernatants were discarded and plates were washed two times gently with 1x PBS. The biofilm was then treated with 0.1% crystal violet stain for 20–30 minutes at room temperature, followed by a wash with distilled water, and allowed to dry at room temperature for 5 hours. The plates were then photographed. For quantification, the bound dye was then re-suspended after 1 h of addition of 1 ml of 33% glacial acetic acid at 25°C. The absorbance was recorded at 570 nm using a plate reader.

### CLSM for biofilm detection

*C. albicans* cells were grown as described above in a 6-well cell culture plate with glass coverslips at 37°C for 24 hr. After 24 hr, the supernatant was discarded and washed with 1x PBS followed by staining with 1% acridine orange for 20 min in dark. Excess stain was removed by washing with 1x PBS, followed by fixation with 4% formaldehyde at 25°C for 30 min. Excess fixative was removed by washing thrice with 1X PBS. Finally, the glass coverslips were placed gently on the glass slide, and images were captured using Leica TCS SP8 confocal scanning system with an excitation wavelength of 483 nm and emission wavelength of 500 to 510 nm bandpass filter.

### Mouse model of systemic candidiasis

The overnight cultures of WT, *pol32*ΔΔ, and *pol32*ΔΔ::*POL32* strains of *C. albicans* were washed thoroughly with sterile distilled water, followed by a saline wash, and finally, the inoculum was prepared in saline. For inoculum preparation, overnight grown strains were washed twice with sterile distilled water followed by washing with PBS and finally dissolved in PBS. The cells were counted using the Neubauer chamber slide in the central area (5 × 5 squares) and 5 × 10^6^ cells/ml cell suspension was prepared for each strain. To reconfirm the inoculum size, CFU analyses for each strains were carried out by spreading a diluted culture of the inoculum on YPD plates. Male BALB/c mice of 5–7 weeks were injected intravenously with 100 μl of 5 × 10^6^ CFU of *C. albicans* cells or saline and survivability were monitored for 30 days. The mice were humanly sacrificed as described earlier.^33^ The kidney, liver, and spleen organs were extracted from each of the mice. Half of these organs were used for CFU determination while the other half of these organs were stored in formalin for tissue sectioning. The mouse kidney paraffin sections were stained with periodic acid Schiff reagents as per manufacturer instructions, and images were captured under a light microscope with 40X magnification. The survival curve was prepared using Graph Pad Prism v8.0 software.

### CFU determination

The kidney, liver, and spleen organs were homogenized in 1x PBS using a tissue homogenizer. An appropriate dilution of the homogenate of each organ was prepared by diluting the sample with 1X PBS and plated on YPD agar plates containing chloramphenicol (50 μg/ml). The colonies were counted for each organ and CFU was determined.

## Supplementary Material

Supplemental Material

## Data Availability

The data that support the findings of this study are available from the corresponding author upon reasonable request. The whole-genome sequence data of *pol32*ΔΔ and WT SC5314 strains can be retrieved from the NCBI database using the accession number PRJNA851971 and PRJN862296.
