# Supplementary material for: Pol32, an accessory subunit of DNA polymerase delta, plays an essential role in genome stability and pathogenesis of Candida albicans
Source: Gut Microbes. 2023 Jan 5;15(1):2163840. doi: 10.1080/19490976.2022.2163840 (PMC9828637; doi:10.1080/19490976.2022.2163840)
Supplement: Supplemental Material [file KGMI_A_2163840_SM0881.pdf]

<u>Supplementary Information</u>

Pol32, an accessory subunit of DNA polymerase delta, plays an essential role in genome stability and pathogenesis of *Candida albicans*

Shraddheya Kumar Patel[1,2], Satya Ranjan Sahu[1,2], Bhabasha Gyanadeep Utkalaja[1,2], Swagata Bose[1,3], and Narottam Acharya[1,*]

[1]Laboratory of Genomic Instability and Diseases, Department of Infectious Disease Biology, Institute of Life Sciences, Bhubaneswar-751023, India.

[2]Regional center of Biotechnology, Faridabad, India.

[3]School of Biotechnology, Kalinga Institute of Industrial Technology, Bhubaneswar-751024, Odisha, India

***Correspondence to:**

Narottam Acharya, Phone: 91-674-2304278, Fax: 91-674-230 0728

E-mail: narottam_acharya@ils.res.in; narottam74@gmail.com

Running title: Essential of role of *POL32*

Key words: DNA replication, Genomics, PCNA, DNA polymerase delta, *Candida*, Candidiasis, Hsp90, azoles, drug resistance, virulence

Supplementary Figure 1. Structural determination of Pol32. Model structures of N terminal region of CaPol32 (1-110 aa) (i), and a superimposed of structures CaPol32 (green) with ScPol32 (Red, 6P1H) (ii) are presented. Model structure of PIP domains of CaPol32 (Red), SpCdc27 (yellow), and ScPol32 (Cyan) was predicted by PEP fold 3.0 and superimposed with the available PIP structure of p68 (Green) without (iii) or with human PCNA structure (iv).

Supplementary Figure 2. *POL32* gene knockout of *C. albicans*. A. Schematic representation of the various constructs and primer positions to generate homozygous deletion of *CaPOL32* gene. **B.** Deletion and integration of *POL32* was confirmed by PCR amplification using orf specific primers. **C**. The cultures of various strains of *C. albicans* and *S. cerevisiae* were grown at 30 °C and absorbance was measured at OD_{600} nm at every 2 hours interval for 14-15 hr. The obtained OD values were plotted using Graph pad prism v8.0.

Supplementary Figure 3. WGS of *Pol32*ΔΔ strain of *C. albicans*. The raw FASTq genome sequence files of our laboratory WT strain SC5314 and *pol32*ΔΔ strain of *C. albicans* were mapped with reference sequence using YMAP. YMAP differentiate between the two haplotypes of a diploid chromosome (A and B) and highlight the copy number variation and aneuploidy using various colors. The grey color indicates heterozygous regions (AB), the cyan color indicates homozygous (AA) regions, and the magenta color indicates homozygous (BB) regions.

Supplementary Figure 4. Gain of indels and SNPs in our laboratory strain. Numbers of indels (i) and SNPs (ii) specifically accumulated in the genome of *pol32*ΔΔ strain of *C. albicans*. Blue color indicates deletion, brown color indicates insertion, grey color indicates homozygous indels, yellow color indicates heterozygous indels, orange color indicates homozygous SNPs, sky blue color indicates heterozygous SNPs, green color indicates transition mutation and maroon color indicates transversion mutation.

Supplementary Figure 5. Semi-quantitative gene expression analysis. Representative gel image of *mRNA* expression of various virulence and hyphal (**A**), and drug resistance (**B**) associated genes determined by RT-PCR, and densitometric analysis of the same was carried out. *GAPDH* was used as an housekeeping gene.

Supplementary Figure 6. Determination of chitin content and berberine or congo-red accumulation assay. Cells were stained with CFW (A), berberine (B), and congo-red (C) and analysed by FACS. The mean fluorescence intensity was given in the table.

Supplementary Figure 7. Mice model of systemic candidiasis. A. Male BALB/c mice of 6-7 weeks of age were inoculated either with WT or *pol32*ΔΔ ($5x10^6$ CFU) *C. albicans* strains intravenously along with

saline control and monitored their survival for 30 days. The survival curve was plotted using Graph pad prism 8.0 software. **B.** The murine kidney, liver, and spleen were collected and fungal burden was measured in all these organs by CFU determination. **C**. The kidney of WT mice (n=2) was stained with PAS staining and images were captured in a light microscope with 40X magnification.

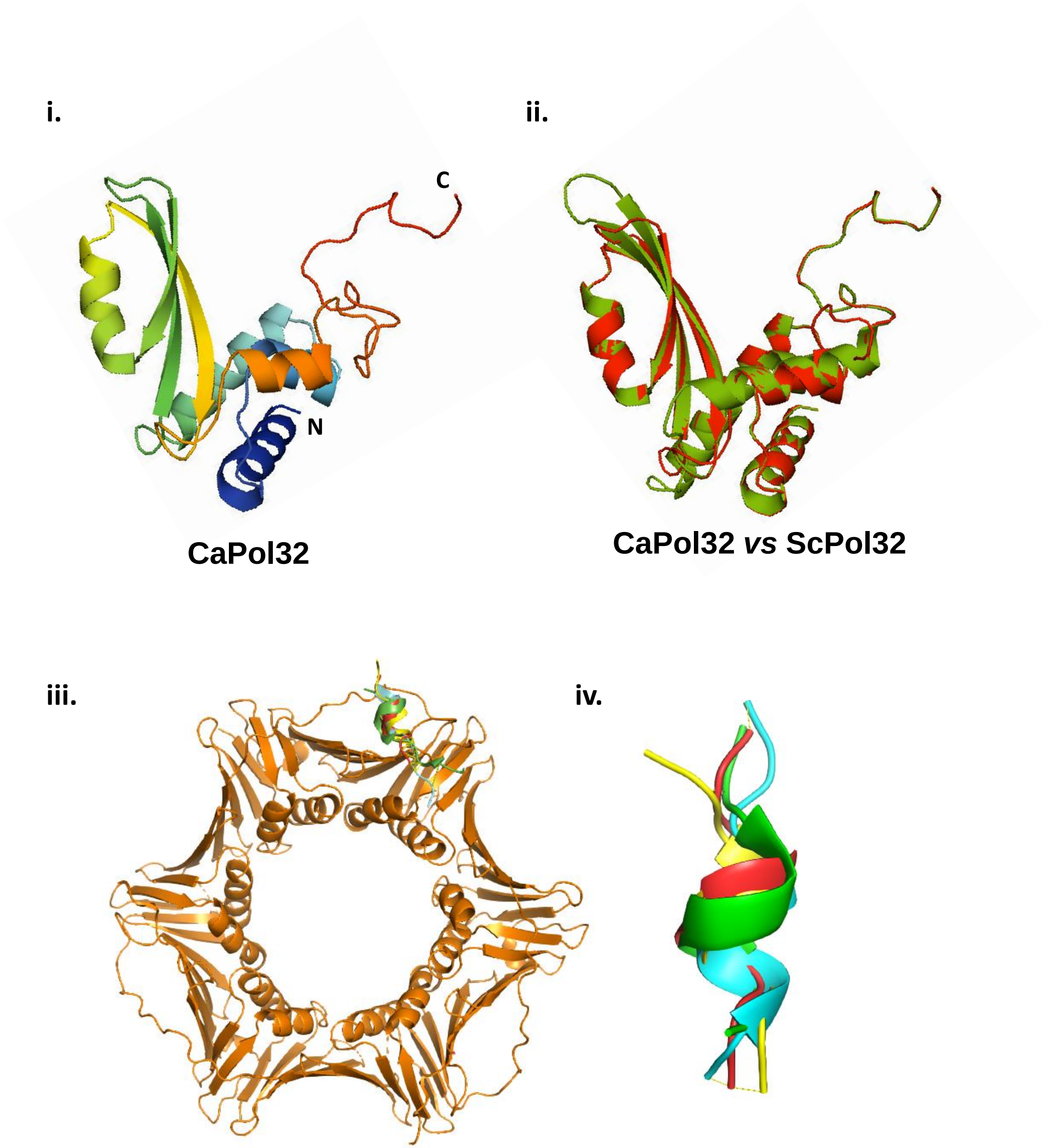

Supplementary Figure 1

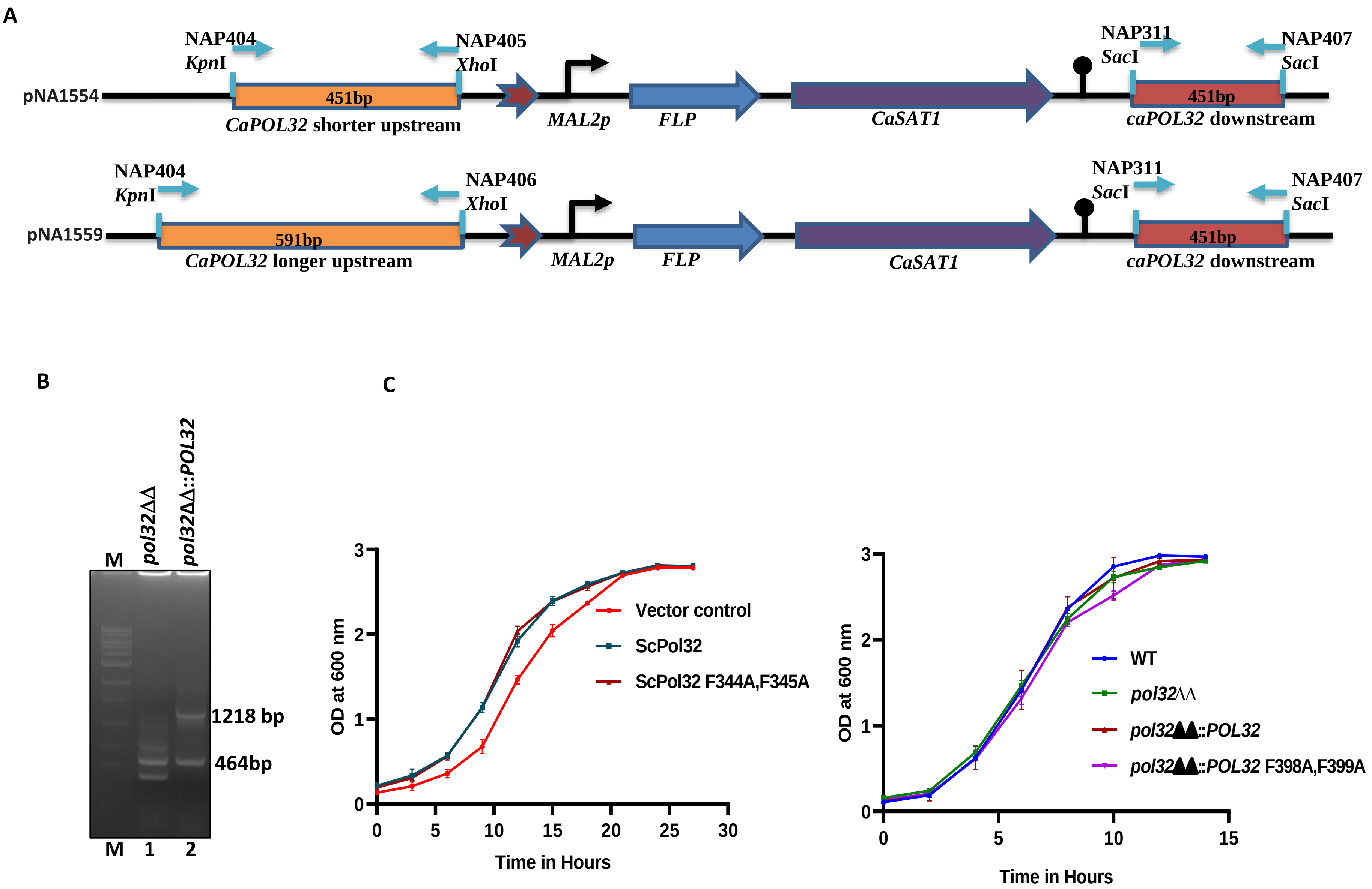

Supplementary Figure 2

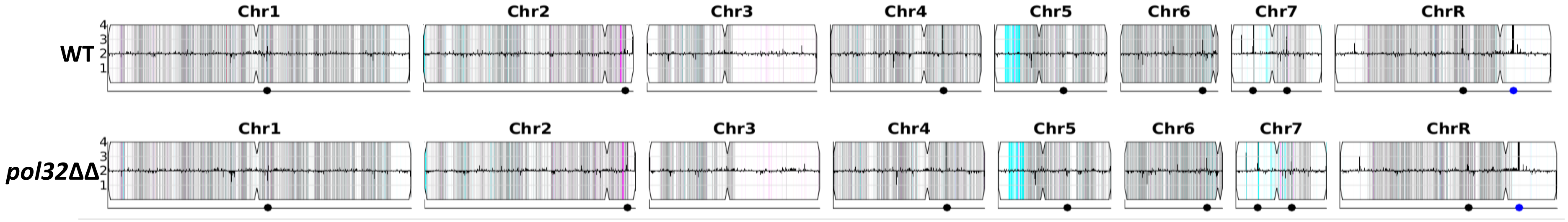

Supplementary Figure 3

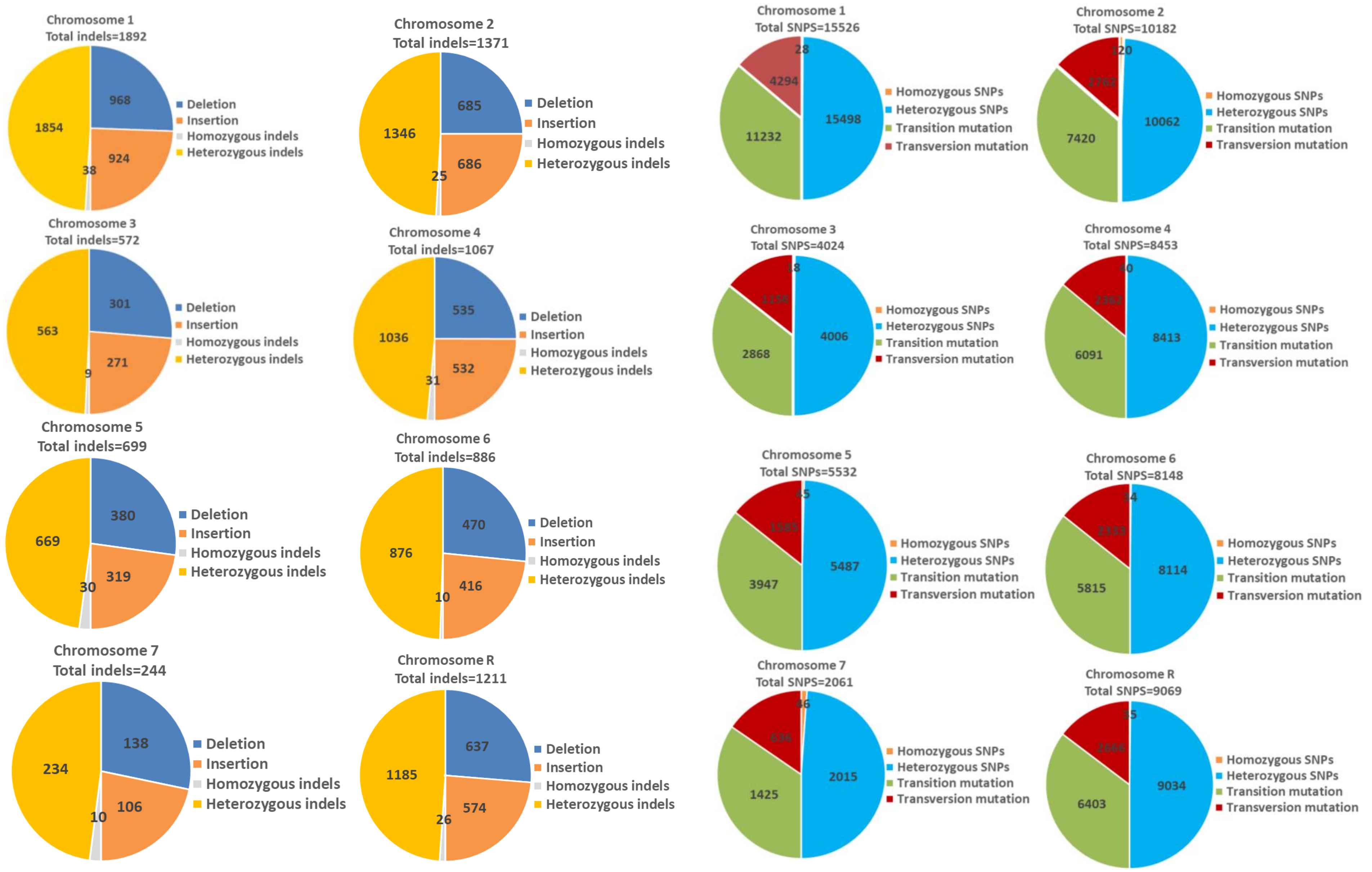

Supplementary Figure 4

A

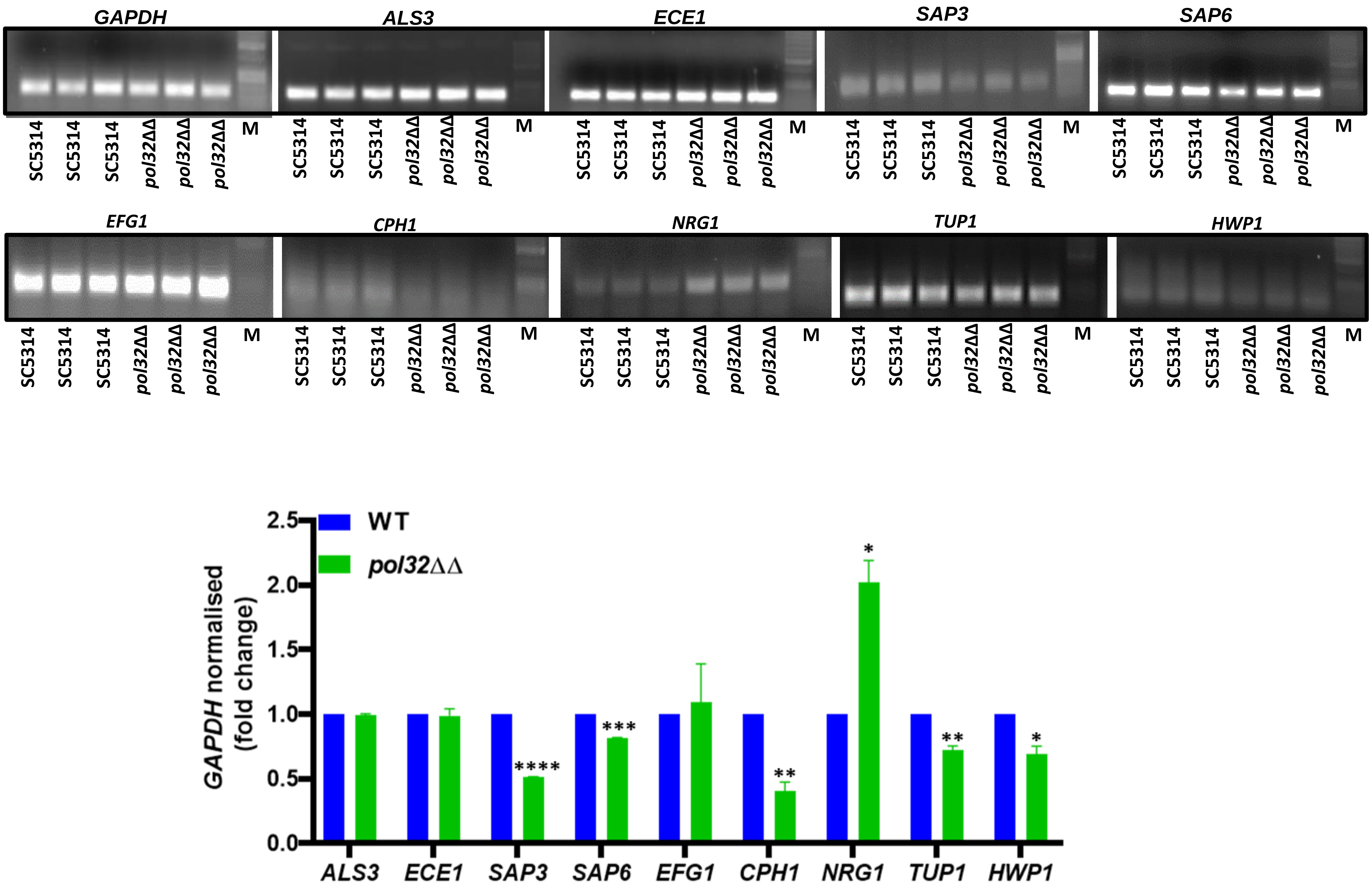

Supplementary Figure 5

B

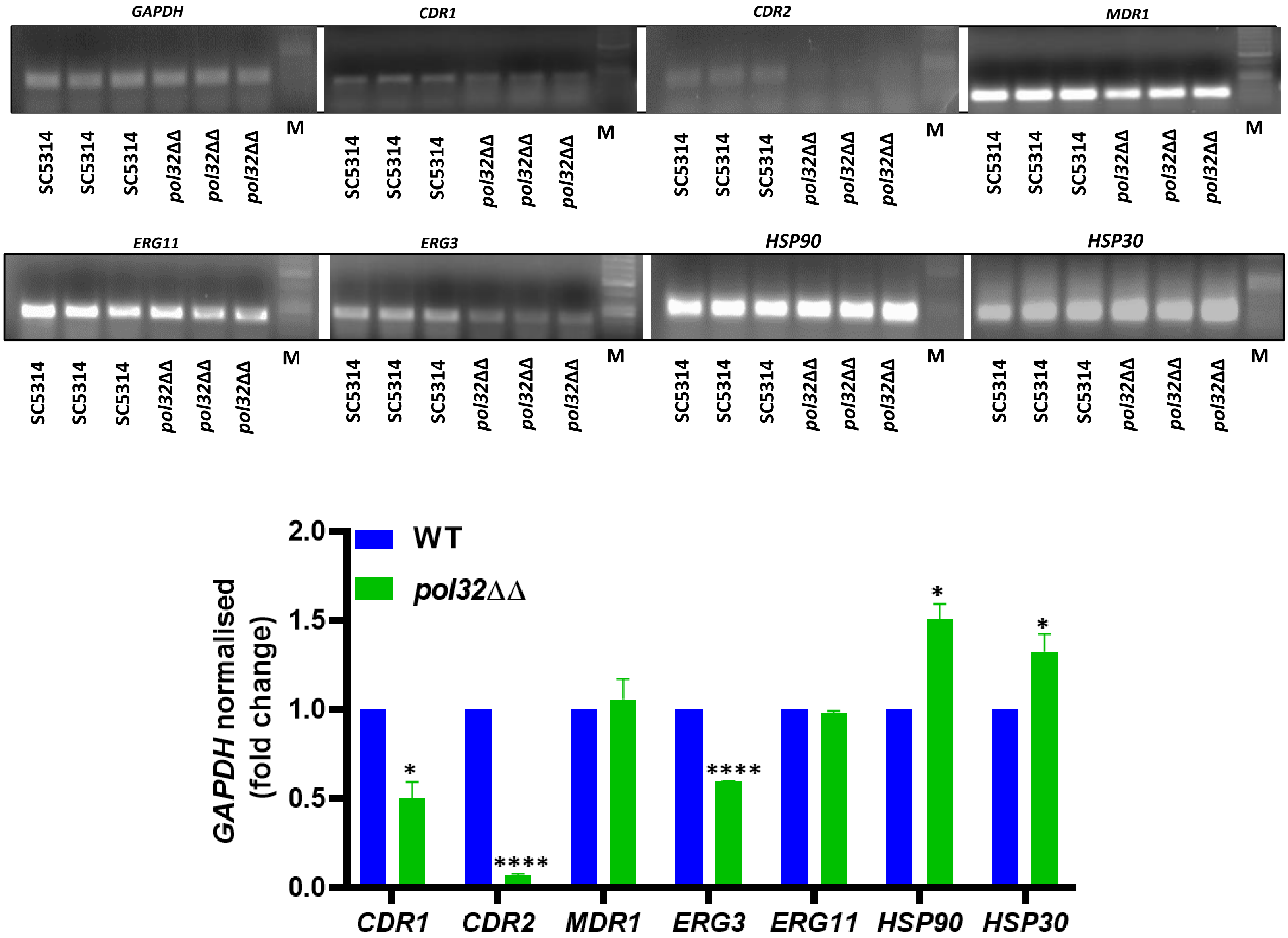

Supplementary Figure 5

A

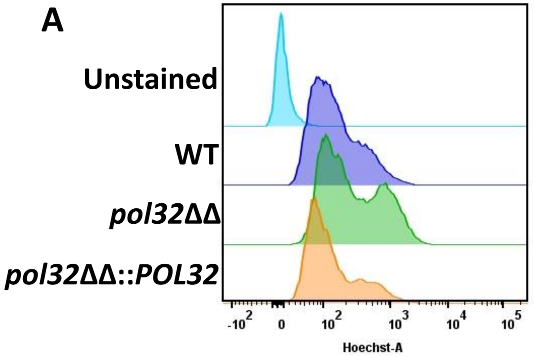

	Sample Name	Subset Name	Count	Mean : Hoechst-A
	CFW_UNSTAINED_001.fcs	CELLS	49838.000	12.649
	CFW_WT 1_002.fcs	CELLS	49766.000	217.879
	CFW_POL32 Delta_006.fcs	CELLS	49332.000	447.691
	CFW_POL32 Re_007.fcs	CELLS	49327.000	212.784

B

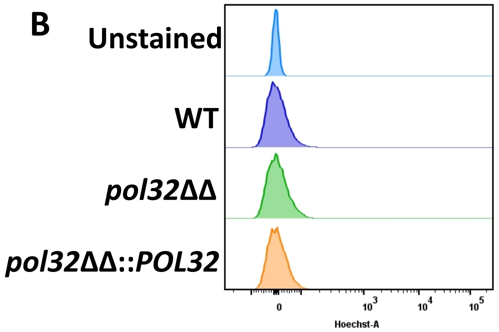

	Sample Name	Subset Name	Count	Mean : Hoechst-A
	BER_unstained_001.fcs	cells	19901.000	-0.693
	BER_WT 2_005.fcs	cells	9809.000	7.616
	BER_pol32_003.fcs	cells	9834.000	8.686
	BER_pol32 re 4_013.fcs	cells	9872.000	6.630

C

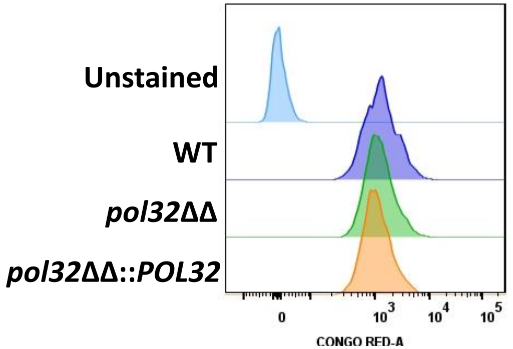

	Sample Name	Subset Name	Count	Mean : CONGO RED-A
	CONGORED_UNSTAINED_001.fcs	CELLS	9708.000	5.191
	CONGORED_WT_003.fcs	CELLS	9324.000	1554.990
	CONGORED_Pol32 Delta_005.fcs	CELLS	9502.000	1433.299
	CONGORED_POL32 Re_002.fcs	CELLS	9387.000	1301.560

Supplementary Figure 6

A

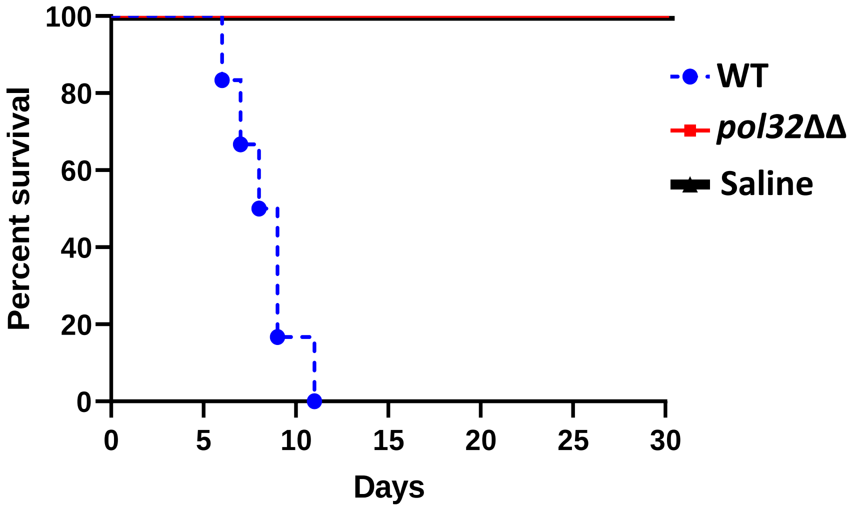

B

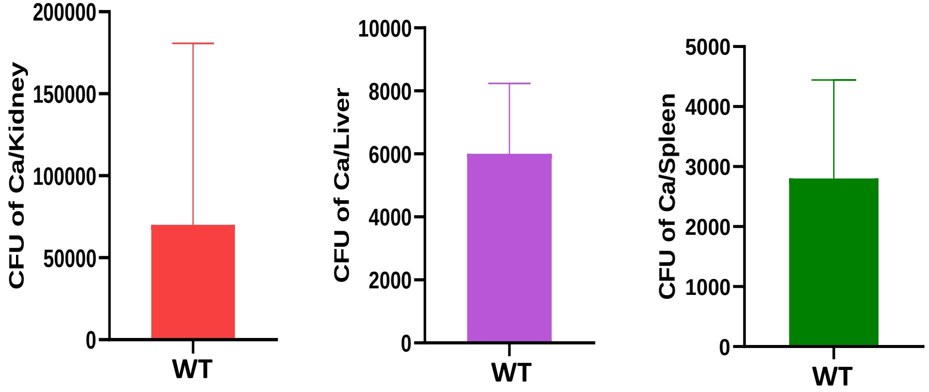

C

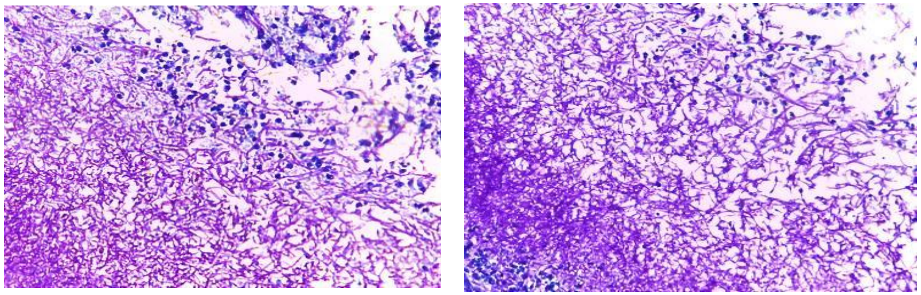

Supplementary Figure 7

Supplementary Table 1: Genetic variations (Indels and SNPs) accumulated in WT strain of *C. albicans*

Chr.no	Total indels	Deletion	Homozygous deletion	Heterozygous deletion	Insertion	Homozygous insertion	Heterozygous insertion		
1	1892	968	14	954	924	24	900		
2	1371	685	13	672	686	12	674		
3	572	301	4	297	271	5	266		
4	1067	535	19	516	532	12	520		
5	699	380	18	362	319	12	307		
6	886	470	8	462	416	2	414		
7	244	138	7	131	106	3	103		
R	1211	637	10	627	574	16	558		
**Total**	**7942**	**4114**	**93**	**4021**	**3828**	**86**	**3742**		
**Chr.no**	**Total SNPs**	**Homozygous SNPS**	**Transition mutation**	**Transversion mutation**	**Heterozygous SNPs**	**Transition mutation**	**Transversion mutation**	**Total transition mutation**	**Total transversion mutation**
1	15526	28	19	9	15498	11213	4285	11232	4294
2	10182	120	77	43	10062	7343	2719	7420	2762
3	4024	18	7	11	4006	2861	1145	2868	1156
4	8453	40	19	21	8413	6072	2341	6091	2362
5	5532	45	26	19	5487	3921	1566	3947	1585
6	8148	34	29	5	8114	5786	2328	5815	2333
7	2061	46	31	15	2015	1394	621	1425	636
R	9069	35	19	16	9034	6384	2650	6403	2666
**Total**	**62995**	**366**	**227**	**139**	**62629**	**44974**	**17655**	**45201**	**17794**

Supplementary Table 2: Repeats region specific accumulation of indels and SNPs in our laboratory WT strain of *C. albicans*

Chr. no	Indels with homo/hetropolymeric repeats	Homozygous indels	Hetrozygous indels	Indels without homo/hetropolymeric repeats	Homozygous indels	Hetrozygous indels
**1**	1170	24	1146	722	14	708
**2**	819	11	808	552	14	538
**3**	338	5	333	234	4	230
**4**	640	20	620	427	11	416
**5**	406	16	390	293	14	279
**6**	513	5	508	373	5	568
**7**	154	6	148	90	4	86
**R**	755	18	737	456	8	448
**Total**	**4795**	**105**	**4690**	**3147**	**74**	**3273**
**Chr.no**	**SNPs with homo/hetropolymeric repeats**	**Homozygous SNPS**	**Hetrozygous SNPS**	**SNPs without homo/hetropolymeric repeats**	**Homozygous SNPs**	**Hetrozygous SNPs**
**1**	7068	16	7052	8458	12	8446
**2**	4530	59	4471	5652	61	5591
**3**	1820	13	1807	2204	5	2199
**4**	3791	17	3774	4662	23	4639
**5**	2421	20	2401	3111	25	3086
**6**	3608	14	3594	4540	20	4520
**7**	957	20	937	1104	26	1078
**R**	4064	23	4041	5005	12	4993
**Total**	**28259**	**182**	**28077**	**34736**	**184**	**34552**

Supplementary Table 3A: Real-time PCR analysis of various genes of WT and *pol32ΔΔ* strains of *C. albicans*. Fold changes in mean of -ΔΔCt values were given.

	WT		*pol32ΔΔ*		
**Gene name**	**Replicate 1**	**Replicate 2**	**Replicate 1**	**Replicate 2**	**T-TEST**
ALS3	1	1	1.0253079	0.7745623	0.508502
ECE1	1	1	0.7708336	0.8331609	0.023888
SAP3	1	1	0.7309944	0.4290872	0.108565
SAP6	1	1	0.5439991	0.1634454	0.076827
EFG1	1	1	1.3132012	1.135971	0.126753
CPH1	1	1	0.6580009	0.9603016	0.333999
NRG1	1	1	1.6932912	1.4740774	0.033501
TUP1	1	1	0.8978614	0.8307234	0.05609
HWP1	1	1	0.5185613	0.2929312	0.034202
CDR1	1	1	0.7991966	0.5395654	0.125741
CDR2	1	1	0.5772788	0.2185022	0.07846
MDR1	1	1	0.5513487	0.2605832	0.055002
ERG11	1	1	0.6720386	0.3700215	0.086672
ERG3	1	1	0.6090152	0.3744086	0.049351
HSP90	1	1	1.9035717	2.1356445	0.012705
HSP30	1	1	1.7933446	1.5070898	0.045194

Supplementary Table 3B: Band intensities of end point PCR products of various genes as depicted in supplementary figure 5A and 5B of WT and *pol32ΔΔ* strains of *C. albicans* were determined by Image J. Fold changes in mean of density with respect to the band of GAPDH were given.

	WT		*pol32ΔΔ*		
**Gene name**	**Replicate 1**	**Replicate 2**	**Replicate 1**	**Replicate 2**	**T-TEST**
ALS3	1	1	0.9836246	1	0.42264973
ECE1	1	1	0.94920792	1.02686725	0.7871505
SAP3	1	1	0.5111574	0.51717255	0.00004
SAP6	1	1	0.81015765	0.81961038	0.00065124
EFG1	1	1	0.88420351	1.30260745	0.6989355
CPH1	1	1	0.35212441	0.45274482	0.0070138
NRG1	1	1	2.13989196	1.90027535	0.01351536
TUP1	1	1	0.69451288	0.74502929	0.00802649
HWP1	1	1	0.65096504	0.73557335	0.01849569
CDR1	1	1	0.56463253	0.43391131	0.01661493
CDR2	1	1	0.07425809	0.06218553	0.000042
MDR1	1	1	0.97721347	1.13633471	0.54951244
ERG11	1	1	0.98820629	0.97106747	0.14067129
ERG3	1	1	0.5966028	0.59278337	0.000022
HSP90	1	1	1.447585	1.5657244	0.01332168
HSP30	1	1	1.25008888	1.39352106	0.04624663
